# Preclinical evaluation of 6-Gingerol in modulating gut microbiota and SCFAs to mitigate *Clostridium difficile*-associated diarrhea in mice

**DOI:** 10.3389/fchem.2025.1635781

**Published:** 2025-08-28

**Authors:** Fu-Zhi Ma, Lin Zhu, Meng Li, Ze-Wei Tang, Xiao-Hong Yu, Cong-En Zhang, Zhi-Jie Ma

**Affiliations:** 1 Department of Pharmacy, Beijing Ditan Hospital, Capital Medical University, Beijing, China; 2 Department of Pharmacy, Beijing Friendship Hospital, Capital Medical University, Beijing, China; 3 College of Traditional Chinese Medicine, Yunnan University of Chinese Medicine, Kunming, Yunnan, China

**Keywords:** 6-Gingerol, *Clostridium difficile*-associated diarrhea, pharmacodynamic evaluation, gut microbiota, short-chain fatty acids (SCFAs)

## Abstract

*Clostridium difficile* associated diarrhea (CDAD) is a growing healthcare concern with limited effective treatments. 6-Gingerol, a major bioactive compound in ginger, exhibits notable antibacterial and anti-inflammatory properties, making it a potential alternative therapy. This study combines *in vitro* and *in vivo* approaches to evaluate its efficacy against CDAD. *In vitro* assays determined the half-maximal inhibitory concentration (IC_50_) and minimum inhibitory concentration (MIC) of 6-Gingerol against *C. difficile*, which were 61.99 μM and 173.3 μM, respectively, indicating direct antibacterial activity. *In vivo*, a mouse model of CDAD was used to assess the therapeutic effects of 6-Gingerol. Outcomes included clinical symptoms, *C. difficile* load, inflammation, intestinal barrier integrity, gut microbiota composition, and short-chain fatty acids (SCFAs) levels. The results showed that in the CDAD mouse model, high-dose 6-Gingerol significantly alleviated CDAD symptoms, reduced *C. difficile* load (*P* < 0.001), improved gut barrier function, and suppressed intestinal inflammation. Although it did not notably increase microbial diversity, 6-Gingerol modulated gut microbiota structure—markedly increasing beneficial bacteria such as *Lactobacillus acidophilus* (*P* < 0.01) and *Bacteroides thetaiotaomicron*, while reducing harmful bacteria including *Klebsiella pneumoniae* and *Proteus mirabilis*. Targeted quantification revealed restored levels of key SCFAs, particularly acetate (*P* < 0.001), butyrate (*P* < 0.01), and valerate (*P* < 0.001), which are closely linked to gut health and recovery from CDAD. In summary, 6-Gingerol exerts therapeutic effects against CDAD through direct inhibition of *C. difficile*, regulation of gut microbiota, restoration of SCFA levels, and protection of the intestinal barrier, highlighting its potential as a novel natural treatment for CDAD.

## 1 Introduction

*C*. *difficile* is a Gram-positive, spore-forming anaerobic bacterium renowned for its resilience in adverse environments, facilitating both intra- and extrahost transmission. *C*. *difficile*-associated diarrhea (CDAD) arises primarily from prolonged or improper antibiotic use, leading to *C. difficile* infection (Martin et al., 2016). Symptoms range from mild diarrhea to severe pseudomembranous colitis, accounting for approximately 25%–30% of antibiotic-associated diarrhea (AAD) cases (McFarland, 2008).

The pathogenesis of CDAD primarily involves antibiotic-induced dysbiosis, which disrupts gut microbiota and weakens colonization resistance against *C. difficile*, allowing extensive colonization and proliferation within the gut (Leffler and Lamont, 2015). *C. difficile* toxins A and B inactivate Rho GTPases, leading to colonocyte death, disruption of intestinal barrier function, and induction of intestinal inflammation, resulting in diarrhea (Chen et al., 2015; Sun et al., 2010; Voth and Ballard, 2005). Key endogenous factors influencing CDAD pathogenesis include gut microbiota homeostasis, gut barrier integrity, and host immune status, while exogenous factors include prolonged or inappropriate antibiotic use and exposure to *C. difficile* (Smits et al., 2016).

In recent years, the pivotal role of short-chain fatty acids (SCFAs) in the pathogenesis and prevention of CDAD has attracted increasing attention. Studies have shown that gut dysbiosis induced by CDAD significantly disrupts intestinal metabolic homeostasis, leading to a marked decrease in the concentrations of SCFAs such as acetate, propionate, butyrate, and valerate (Gregory et al., 2021). SCFAs not only provide a primary energy source for intestinal epithelial cells (IECs), but also regulate epithelial cell function, gut motility, barrier integrity, and host immune responses (Martin-Gallausiaux et al., 2021). Supplementation with SCFAs, particularly acetate and butyrate, has been shown to improve CDAD outcomes (Ouyang et al., 2022).

Recent experimental studies have further revealed that modulating SCFAs metabolism or supplementing specific SCFAs can directly suppress *C. difficile* growth and infection. For instance, restoration of valerate levels through fecal microbiota transplantation (FMT) inhibits recurrent infection (McDonald et al., 2018), while acetate promotes IL-22 secretion by ILC3s to enhance mucosal immunity (Fachi et al., 2020; Fachi et al., 2025). Exogenous butyrate has also been shown to inhibit multiple *C. difficile* strains (Pensinger et al., 2023; Pensinger et al., 2024). Collectively, these findings indicate that SCFAs play a central role in maintaining intestinal homeostasis and host defense, and represent promising targets for the prevention and treatment of CDAD.

Vulnerable populations, such as hospitalized patients, the elderly, children, and individuals with compromised immune systems and reduced gut barrier function, are particularly susceptible to CDAD (Czepiel et al., 2019; Smits et al., 2016). To accurately simulate the clinical condition of immunosuppressed patients and reflect the sensitivity to *C. difficile* infection and subsequent pathophysiological changes, this study employs a dexamethasone-induced immunosuppressed mouse model secondary to CDAD (Kim et al., 2016; Sun et al., 2011). Globally, particularly in China, the incidence and mortality rates of CDAD have escalated due to rampant antibiotic misuse, posing a serious public health threat (Cheng et al., 2016; Tang et al., 2016; Wen et al., 2023). Current guidelines for treating CDAD recommend antibiotics such as metronidazole and vancomycin, but recurrence rates are high, ranging from 20% to 30% (Johnson and Gerding, 2019; Stevens et al., 2017). For patients with multiple recurrences, the risk can escalate to 65% (Gough et al., 2011). Additionally, *C. difficile* has shown resistance trends to fidaxomicin (Hvas et al., 2019). In recent years, modern biological therapies, such as fecal microbiota transplantation (FMT), monoclonal antibodies, and microbiota-based therapeutics, have been increasingly used to treat CDAD, but their efficacy and safety require further validation (Ivarsson et al., 2019; Oksi et al., 2020).

Our research group previously demonstrated that Shengjiang Xiexin Decoction has therapeutic effects on CDAD in mice, including inhibition of *C. difficile* growth, restoration of gut microbiota homeostasis, and enhancement of gut barrier function (Cui et al., 2023; Yu et al., 2024). The decoction includes ginger and dried ginger, with ginger being the principal component. 6-Gingerol, the major active ingredient in both ginger and dried ginger. Our studies confirmed that both fresh ginger and dried ginger alone effectively ameliorate gut microbiota, enhance gut barrier function, and treat antibiotic-associated diarrhea (Ma et al., 2020). Given its significant efficacy, 6-Gingerol likely contributes to these therapeutic effects against CDAD.

Further analyses reveal that 6-Gingerol exhibits anti-inflammatory, analgesic, antibacterial, antitumor, antioxidant, gut barrier repair, and immunomodulatory properties (Li et al., 2017). Yanli Li et al. found that 6-Gingerol protects the intestinal mucosal barrier by inhibiting the NF-κB signaling pathway (Li et al., 2017). Xiao-Xuan Guo et al. discovered that 6-Gingerol prevents lipopolysaccharide-induced gut barrier damage and liver injury in mice (Guo et al., 2022). Kuei-Wen Chang et al. revealed that 6-Gingerol modulates the pro-inflammatory response in dextran sulfate sodium (DSS)-treated Caco-2 cells and experimental colitis in mice by activating adenosine monophosphate-activated protein kinase (AMPK) (Chang and Kuo, 2015). Collectively, these findings suggest that 6-Gingerol may effectively ameliorate CDAD and restore gut microbiota and metabolic homeostasis. This study investigates the intervention effects of 6-Gingerol on CDAD, examining pharmacodynamic indices, intestinal inflammation, gut barrier function, gut microbiota structure, and SCFAs levels, providing scientific insights for CDAD prevention and treatment strategies.

## 2 Materials and methods

### 2.1 Chemical reagents

The antibiotics metronidazole (S17079, purity ≥98%), vancomycin (S17059, 900 mcg/mg), gentamicin (S17024, 590 µ/mg), clindamycin (S63729, purity ≥98.5%), and kanamycin (S17025, 750 µ/mg) were procured from Shanghai Yuanye Bio-Technology Co., Ltd. Colistin (MB1064, ≥19,000 IU/mg) was obtained from Dalian Meilun Biotechnology Co., Ltd., and dexamethasone (ST1254, purity ≥99%) from Shanghai Beyotime Biotechnology Co., Ltd. 6-Gingerol (PU0924-0200, purity ≥98.5%) was purchased from Chengdu Push Bio-Technology Co., Ltd.

### 2.2 *C. difficile* strain

*C*. *difficile* strain 630 (ATCC BAA-1382) was provided by ATCC. The strain was cultured on Columbia blood agar plates under standard anaerobic conditions at 37°C for 7 days. *C. difficile* cells were harvested from the culture medium using a sterile loop and resuspended in phosphate-buffered saline (PBS). To isolate *C. difficile* spores, the suspension was heat-treated at 65°C for 20 min to eliminate vegetative cells. The treated suspension was centrifuged at 3,000 × g, washed 2-3 times with PBS, and the supernatant discarded. The resulting pellet was resuspended in liquid medium and stored at −80°C for future use.

### 2.3 The *in vitro* antibacterial activity of 6-Gingerol

The prepared *C. difficile* suspension was adjusted to 0.5 McFarland standard using PBS and inoculated into a 96-well plate. For the experimental group, each well received 180 μL of liquid culture medium and 20 μL of *C. difficile* suspension, while the control group received 180 μL of liquid culture medium and 20 μL of PBS. During incubation, OD600 values were measured every 2 h to monitor bacterial growth dynamics and generate a growth curve.

After constructing the growth curve, working solutions of 6-Gingerol and fidaxomicin were prepared using anaerobic culture medium. The final concentrations of 6-Gingerol were set at 500 μM, 400 μM, 200 μM, 100 μM, 75 μM, 50 μM, 25 μM, 10 μM, and 5 μM. The final concentrations of fidaxomicin were set at 4 μM, 2 μM, 1 μM, 0.5 μM, 0.25 μM, 0.125 μM, 0.0625 μM, 0.03125 μM, 0.015625 μM, and 0.0078125 μM. For each concentration, 180 μL of the prepared working solution was added into individual wells of a 96-well plate, with three replicates per concentration. Then, 20 μL of freshly prepared *C. difficile* suspension was added to each well. For the blank control group, 180 μL of the corresponding drug solution and 20 μL of PBS were added to evaluate potential interference of the compounds with the OD readings.

OD600 values were measured at specific time points during the logarithmic growth phase to evaluate the effect of 6-Gingerol on *C. difficile* growth and to determine its minimum inhibitory concentration (MIC) and half-maximal inhibitory concentration (IC50). MIC: The OD600values were modelled using a modified non-linear regression method based on an F-test (GraphPad Prism 9.0) (Lepe et al., 2013). OD600 data (bacterial growth) were adjusted by the Gompertz equation to calculate the MIC and confidence intervals (CIs) directly, and were not based on the inflection point. IC50: Using GraphPad Prism, the “log (inhibitor) vs. response—Variable slope (four parameters)” model was employed to perform dose-response curve fitting. This method conducts regression analysis on the relationship between the logarithm of the inhibitor concentration and the OD600 response, thereby accurately determining the IC50 value of 6-Gingerol and providing a quantitative basis for evaluating its antimicrobial activity.

### 2.4 Experimental animals and design

Specific-pathogen-free (SPF) male C57BL/6 mice, aged 5 weeks and weighing 20 ± 2 g, were obtained from SPF (Beijing) Biotechnology Co., Ltd. (License No. SCXK (Jing) 2019-0010). The mice were housed in an SPF-grade laboratory with controlled temperature (20°C–26°C), humidity (40%–70%), and a 12-h light/dark cycle. Standard rodent feed and water were provided *ad libitum*. All animal experiments were conducted in accordance with ethical guidelines approved by the Animal Ethics Committee of Beijing Friendship Hospital, Capital Medical University.

After 1 week of acclimatization, 40 mice were weighed and randomly assigned to five groups (n = 8/group): control (CON), model (MOD), fidaxomicin (FID), low-dose 6-Gingerol (GL), and high-dose 6-Gingerol (GH). Randomization was performed using the RAND function in Excel. All groups were maintained under normal conditions.

The experimental protocol is illustrated in Figure 2A. From day −6 to day −3, mice in the CON group received normal drinking water, while those in the MOD, GL, and GH groups were given water containing a combination of antibiotics and dexamethasone (kanamycin: 0.4 mg/mL; vancomycin: 0.045 mg/mL; metronidazole: 0.215 mg/mL; colistin: 850 U/mL; gentamicin: 0.035 mg/mL; dexamethasone: 0.1 mg/mL). From day −3, all mice resumed normal drinking water. On day −1, mice, except those in the CON group, were intraperitoneally injected with 10 mg/kg clindamycin; the CON group received an equivalent volume of saline. On day 0, all mice, except those in the CON group, were orally administered 500 μL of *C. difficile* spores (10^5^ CFU/mouse); the CON group received an equivalent volume of saline. From day 1 to day 7, drug treatments were administered daily at 9:00 a.m.: the FID group received 30 mg/kg fidaxomicin, the GH group received 100 mg/kg 6-Gingerol, the GL group received 50 mg/kg 6-Gingerol (Alhamoud et al., 2023; Chang and Kuo, 2015; Guo et al., 2022), and the CON and MOD groups received equivalent volumes of saline. The body weight and general condition of all mice were monitored and recorded. On day 8, all mice were euthanized, and colonic tissues were collected for subsequent analyses.

### 2.5 Clinical symptom assessment and fecal consistency evaluation

Clinical symptoms of CDAD were assessed based on the criteria outlined in Table 1 (Fachi et al., 2020).

**TABLE 1:** Clinical scoring.

Category	Score			
	0	1	2	3
Activity	Normal	Alert/slow moving	Lethargic/shaky	Inactive unless prodded
Posture	Normal	Back slanted	Hunched	Hunched/nose down
Coat	Normal	Piloerection	Rough skin	Very ruffled/puff/ungroomed
Diarrhea	Normal	Soft stool/discolored (yellowish)	Wet stained tail/mucous ± blood	Liquid/no stool (ileus)
Eyes/nose	Normal	Squinted/half closed	Squinted/discharge	Closed/discharge

Fecal consistency was visually assessed and categorized into three grades: (1) Formed: feces retained shape and were brown (score 1); (2) Semi-formed/soft: feces did not retain shape and were yellow (score 2); (3) Liquid: easily poured and yellow (score (Figure 2D).

### 2.6 Real-time quantitative PCR analysis

We utilized RT-qPCR to quantify *C. difficile* burden by detecting the *toxin B* gene. Total DNA from mouse feces was extracted using the E.Z.N.A.^®^ Stool DNA Kit (D4015-01, OMEGA), and the DNA was stored at −20°C. Real-time quantitative PCR (RT-qPCR) was performed using a 25 μL reaction mixture containing 12.5 μL Premix Ex Taq TM (RR390, TAKARA), 0.5 μL Rox reference dye II (AK60353A, TAKARA), 2.5 μL forward and reverse primers with TaqMan probe (1927827100, Sangon Biotech), and 2 μL DNA. The assay was run on a 7500 FAST Real-Time PCR System (Thermo Fisher Scientific) with a two-step PCR amplification protocol: initial denaturation at 95°C for 30 s, followed by 40 cycles of 95°C for 3 s and 50°C for 30 s. Primer and probe sequences were: stcdb-f (5′-3′: ATATCA GAG ACT GAT GAG), stcdb-r (5′-3′: TAGCAT ATT CAG AGA ATA TTG T), and stcdb-p (5′-3′: FAM-C TGG AGA ATC TAT ATT TGT AGA AAC TG-BHQ).

Distal colon tissues were collected immediately after euthanasia, rinsed with pre-cooled PBS, and preserved in RNAlater (AM7021, Invitrogen), followed by storage at −20°C. Total RNA was extracted, and quantitative real-time PCR was performed to assess the expression of IL-1β, TNF-α, Occludin, and ZO-1, using GAPDH as the internal reference gene. Total RNA was extracted using Trizol (391107, Ambion) and reverse transcribed to cDNA using a reverse transcription kit (0202010531, LABLEAD). RT-qPCR was performed with a two-step amplification protocol: initial denaturation at 95°C for 5 min, followed by 40 cycles of 95°C for 10 s and 60°C for 30 s. The reaction mixture included 10 μL SYBR Green Master Mix, 1 μL each of forward and reverse primers, 2 μL DNA template, and 6 μL Nuclease-Free Water. Primer sequences used were: IL-1β (F: 5′-3′: TGT​GAA​ATG​CCA​CCT​TTT​GA, R: 5′-3′: GTC​AAA​GGT​TTG​GAA​GCA​G), TNF-α (F: 5′-3′: CTC​CAG​GCG​GTG​CCT​ATG​T, R: 5′-3′: GAA​GAG​CGT​GGT​GGC​CC), Occludin (F: 5′-3′: TAC​TGT​GTG​GTT​GAT​CCC​CAG, R: 5′-3′: GAT​AAT​CAT​GAA​CCC​CAG​GAC), ZO-1 (F: 5′-3′: CCA​CTC​TTC​CAG​AAC​CGA​AAC​CTG, R: 5′-3′: TTT​CAT​GCT​GGG​CCT​AAG​TAT​CCC), GAPDH (F: 5′-3′: GGC​AAG​GTC​ATC​CCA​GAG​CTG, R: 5′-3′: ATC​CAC​GAC​GGA​CAC​ATT​GGG).

### 2.7 Histological analysis

Colonic tissues were fixed in 4% paraformaldehyde, paraffin-embedded, sectioned, and stained with hematoxylin and eosin (H&E) for pathological examination. Goblet cell counts were determined using Alcian Blue staining.

For immunohistochemical analysis, sections were placed in citrate antigen retrieval buffer (pH 6.0) for antigen retrieval and blocked with 3% hydrogen peroxide to inhibit endogenous peroxidase activity. Primary antibodies included rabbit anti-MPO (22225-1-AP, Proteintech) and rabbit anti-MUC2 (27675-1-AP, Proteintech). Negative expression was indicated by blue staining, and positive expression by brown staining.

For immunofluorescence analysis, sections were boiled in EDTA/citrate buffer for antigen retrieval and blocked with PBS containing 3% bovine serum albumin (BSA). Primary antibodies were rabbit anti-ZO-1 (21773-1-AP, Proteintech) and rabbit anti-PCNA (10205-2-AP, Proteintech). Secondary antibodies were Anti-rabbit IgG (H + L), F (ab′)2 Fragment (4412S, CST). Nuclei were stained with DAPI (D1306, Invitrogen), which appeared blue, while FITC-conjugated secondary antibodies appeared green. Semi-quantitative analysis of immunofluorescence and immunohistochemistry was performed using HALO software (Indica Labs, United States).

### 2.8 16S rRNA gene sequencing of colonic contents

16S rRNA sequencing targeted five regions (V2, V3, V5, V6, V8) through multiplex PCR amplification and sequencing. PCR products were purified, pooled, and sequenced on an Illumina NovaSeq 6000 platform with paired-end 150 bp reads. Negative extraction controls and no-template PCR controls were included throughout the DNA extraction and library preparation processes to monitor for potential contamination. Data were demultiplexed based on PCR and Barcode sequences, trimmed, and processed using FLASH2 (Version 2.2.00) to generate raw tags. Clean tags were obtained through stringent quality filtering, and chimeric sequences were removed using QIIME (Version 2.0) quality control pipelines. Effective tags were used for species annotation and abundance analysis, including alpha diversity for within-group microbial complexity and beta diversity for inter-group compositional differences. Cluster analysis and species composition comparisons were conducted to identify differences among groups, and statistical methods at various taxonomic levels were applied to analyze bacterial community structure.

### 2.9 Targeted quantitative analysis of fecal short-chain fatty acids

At the end of the 6-Gingerol treatment, fecal samples from the CON, MOD, and GH groups were subjected to targeted quantitative analysis of short-chain fatty acids using ultra-performance liquid chromatography-mass spectrometry (UPLC-MS). This analysis quantified acetate, propionate, isobutyrate, butyrate, valerate, isovalerate, hexanoic acid, and heptanoic acid. Detailed methods are provided in the supplementary materials, including standard curves (Supplementary Figure S5) and total ion chromatograms (TIC) of samples (Supplementary Figure S6).

### 2.10 Data analysis

Statistical analyses were performed using SPSS 26.0 software. Results are expressed as mean ± standard deviation (SD). One-way ANOVA was used to determine statistical significance, and non-parametric Kruskal-Wallis tests were applied where appropriate. Differences were considered statistically significant at *P* < 0.05.

## 3 Results

### 3.1 Determination of the MIC and IC50 of 6-Gingerol against *Clostridium difficile*

**FIGURE 1:**
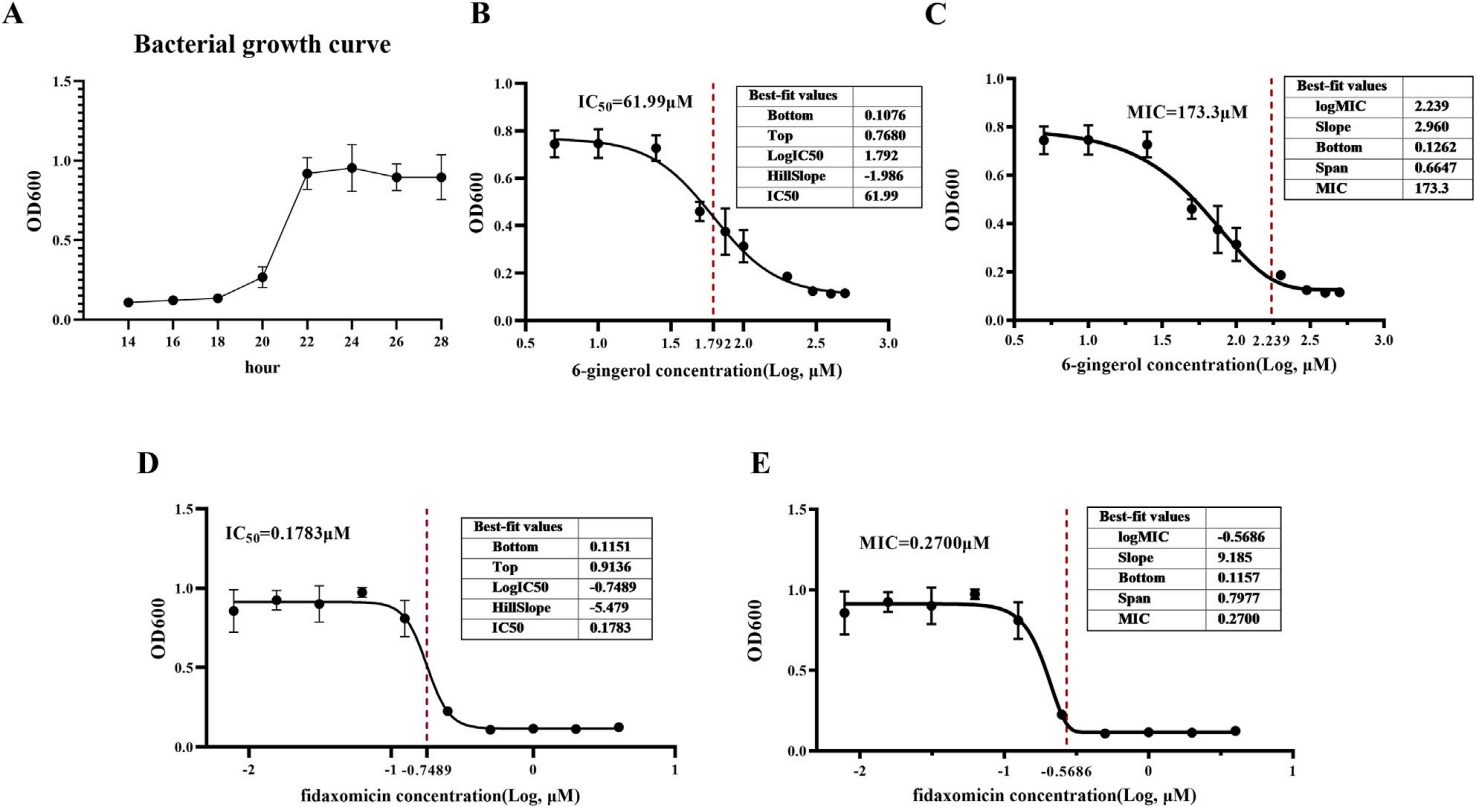
The *in vitro* antibacterial activity of 6-gingerol. **(A)** *Clostridium difficile* growth curve. **(B)** IC50 of 6-gingerol against *Clostridium difficile*. **(C)** MIC of 6-gingerol against *Clostridium difficile*. **(D)** IC50 of fidaxomicin against *Clostridium difficile*. **(E)** MIC of fidaxomicin against *Clostridium difficile*. Data are presented as mean ± standard deviation from three independent replicates. MIC was calculated using non-linear regression based on the Gompertz model and F-test, and IC_50_ was calculated using four-parameter logistic regression (“log [inhibitor] vs. response–variable slope”) in GraphPad Prism 9.0.

This study evaluated the inhibitory effects of various concentrations of 6-Gingerol and the positive control drug fidaxomicin on the growth of *Clostridium difficile*, aiming to determine their minimum inhibitory concentration (MIC) and half-maximal inhibitory concentration (IC_50_). As shown in Figure 1A. *C. difficile* exhibited a typical bacterial growth curve from 14 to 28 h, with OD_600_ increasing from approximately 0.1 to around 1.0 and plateauing after 24 h. Since the logarithmic growth phase occurs between 20 and 22 h, the 22-h time point was selected for MIC and IC_50_ determination to ensure data consistency and comparability.

As the concentration of 6-Gingerol increased, the growth of *C. difficile* was significantly inhibited, as reflected by a concentration-dependent decline in OD_600_ values (Figures 1B,C). At lower concentrations, bacterial growth was still partially maintained; however, higher concentrations led to a marked reduction in OD_600_, indicating potent antibacterial activity. Using a “log (inhibitor) vs. response–Variable slope (four parameters)” model for curve fitting, the IC_50_ of 6-Gingerol was calculated to be approximately 61.99 μM. The MIC, determined using a modified non-linear regression method based on the F-test, was approximately 173.3 μM, at which complete inhibition of bacterial growth was observed. In comparison, the positive control fidaxomicin demonstrated significantly stronger antibacterial activity under the same conditions, with an IC_50_ of 0.1783 μM and an MIC of 0.2700 μM (Figures 1D,E).

In summary, 6-Gingerol exhibited a clear concentration-dependent inhibitory effect on the growth of *C. difficile*, with complete inhibition achieved at higher concentrations. Although its antibacterial potency was lower than that of fidaxomicin, 6-Gingerol, as a natural bioactive compound, shows promising potential in combating *C. difficile* infection and lays a solid foundation for further investigation into its antibacterial mechanisms and clinical applications.

### 3.2 Efficacy of 6-Gingerol in mitigating CDAD symptoms

**FIGURE 2:**
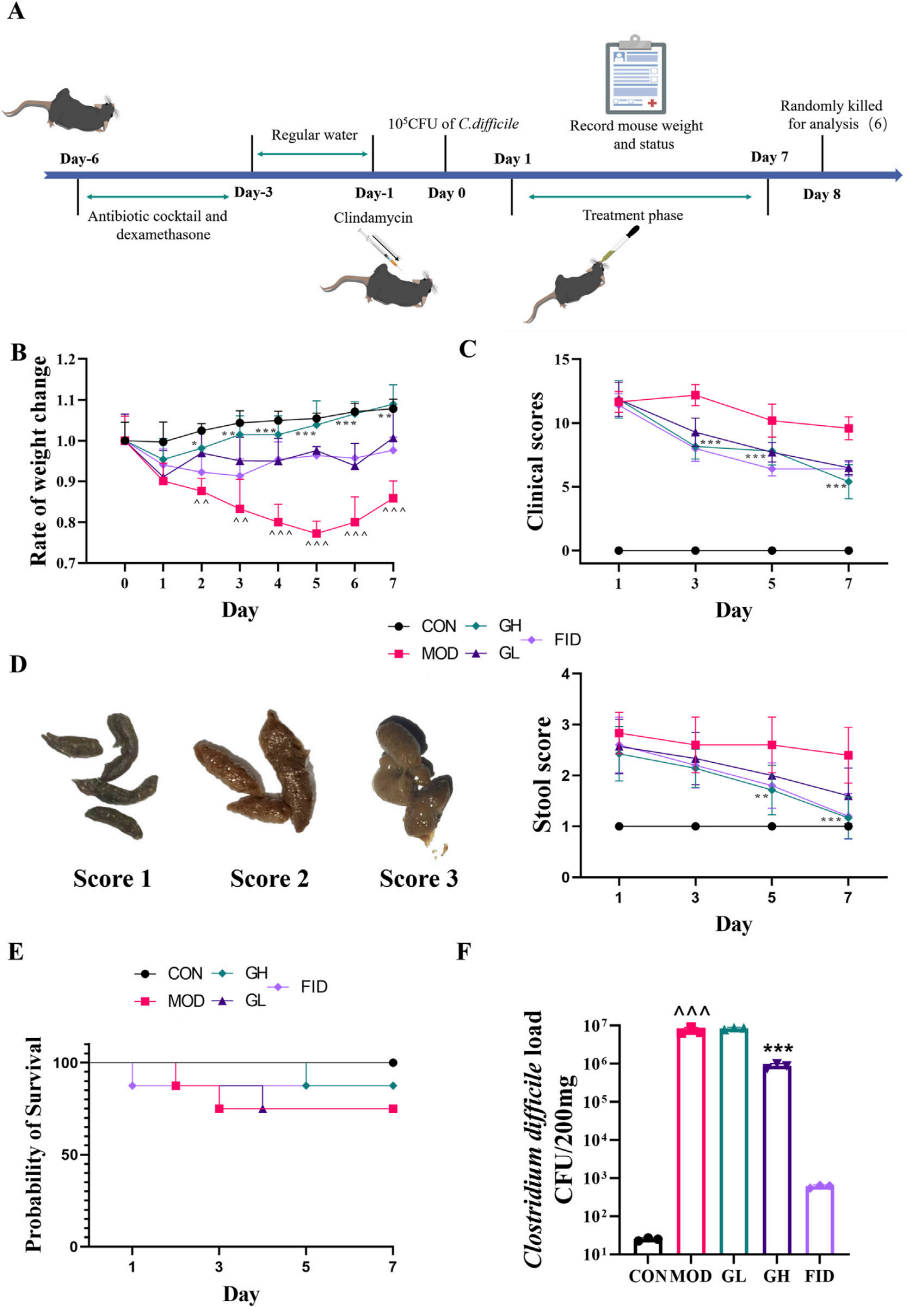
The ameliorative effects of 6-Gingerol on CDAD. **(A)** Schematic of the animal model and experimental timeline. **(B)** Body weight change rates. **(C)** Clinical scores. **(D)** Evaluation and classification of fecal consistency using a three-grade visual scale. **(E)** Mortality rates. **(F)** Fecal *C. difficile* load on day 7. Values are expressed as mean ± standard deviation. n = 5/group for **(A–D)**; n = 8/group for **(E)**. n = 3/group for **(F)**. Statistical comparisons were performed using one-way ANOVA. * indicates a significant difference between the MOD and GH groups, *P < 0.05, **P < 0.01, ***P < 0.001. ^ indicates a significant difference between the MOD and CON groups, ^*P* < 0.05, ^ ^*P* < 0.01, ^ ^ ^*P* < 0.001.

Upon establishing the animal model, we monitored the body weight, clinical symptoms, fecal consistency, and mortality of the mice (Figure 2A). Mice in the CON group exhibited a continuous increase in body weight, whereas those in the other groups experienced a rapid decline in body weight on the first day following gavage with *C. difficile* spores. In the MOD group, body weight continued to decrease until day five, followed by a gradual increase over the next 2 days. During the treatment phase, the GH, GL, and FID groups showed an upward trend in body weight, with the GH group displaying a rate of weight change closer to the CON group. Significant differences in body weight were observed between the GH and GL groups compared to the MOD group from day two to the end of the experiment, with the most pronounced difference on day five (*P* < 0.001). These findings indicate that 6-Gingerol significantly restored the body weight of CDAD mice (Figure 2B).

Clinical symptoms and fecal consistency serve as direct indicators of CDAD severity. We employed scoring methods to evaluate these parameters, referencing Figure 2D for fecal consistency and Table 1 for clinical symptoms. Throughout the experiment, clinical and fecal parameters in the CON group remained normal. In contrast, the MOD group consistently showed higher scores than the treatment groups. After treatment, all groups exhibited some degree of recovery from CDAD symptoms. By day seven, fecal consistency in the GH group had returned to normal levels (score 1), with significant improvements in clinical status compared to the MOD group. Similarly, the GL group showed notable recovery. These data demonstrate that 6-Gingerol effectively ameliorates clinical symptoms and fecal consistency in CDAD mice.

On the final day of treatment, fecal samples were collected for quantification of *C. difficile* load. As shown in Figure 2E, the MOD group exhibited a significantly higher *C. difficile* load than the CON group, confirming successful model establishment. The FID group showed a *C. difficile* load comparable to the CON group, indicating effective pathogen suppression. Notably, the GH group demonstrated a significantly lower *C. difficile* load than both the GL and MOD groups, while no significant difference was observed between the GL and MOD groups. These findings indicate that the marked reduction in *C. difficile* load achieved by high-dose 6-Gingerol treatment played a critical role in mitigating CDAD.

### 3.3 6-Gingerol reduces colonic inflammation in CDAD

**FIGURE 3:**
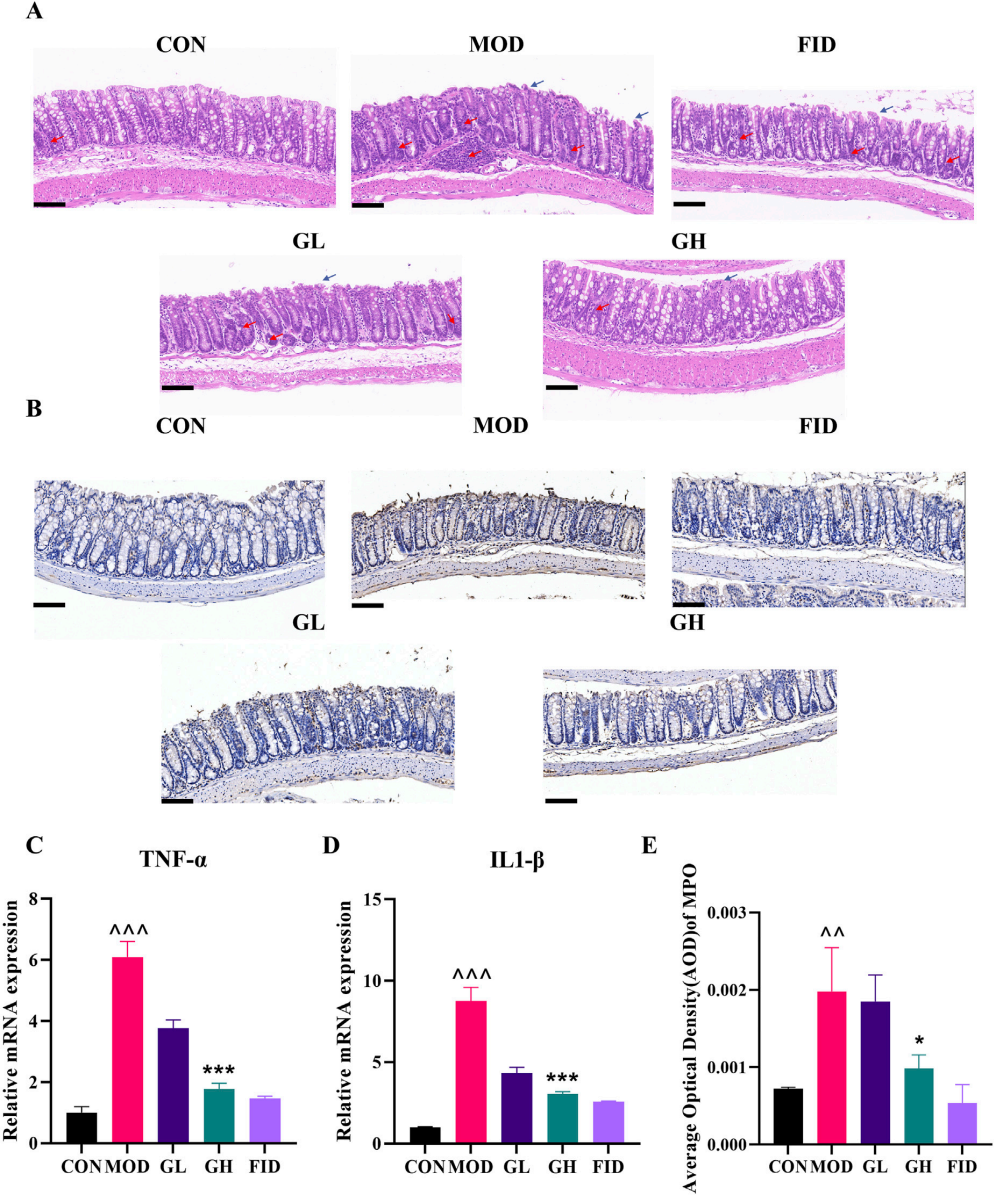
The restorative effects of 6-Gingerol on intestinal inflammation in CDAD mice. **(A)** Histopathological images of H&E-stained colonic samples. Blue arrows: shed mucosal epithelial cells; red arrows: inflammatory cell infiltration. **(B)** Immunohistochemical analysis of MPO expression in colonic tissues. **(C,D)** RT-PCR analysis of TNF-α **(C)** and IL-1β **(D)**. **(E)** Semi-quantitative analysis of MPO immunohistochemistry. Original magnification: 200x, scale bar: 100 μm. Values are expressed as mean ± standard deviation, n = 3/group. Statistical comparisons were performed using one-way ANOVA. * indicates a significant difference between the MOD and GH groups, *P < 0.05, **P < 0.01, ***P < 0.001. ^ indicates a significant difference between the MOD and CON groups, ^*P* < 0.05, ^ ^*P* < 0.01, ^ ^ ^*P* < 0.001.

Histopathological examination of H&E-stained colonic samples (Figure 3A) revealed intact and closely packed mucosal epithelial cells with minimal inflammatory infiltration in the CON group. Conversely, the MOD group exhibited extensive inflammatory cell infiltration into the submucosa, epithelial cell shedding, and severe disruption of the intestinal barrier. The FID group showed reduced inflammatory cell infiltration in the lamina propria and tightly arranged mucosal epithelial cells with only minimal shedding. In both 6-Gingerol treatment groups, colonic tissue pathology was less severe than in the MOD group, with the GH group showing significantly reduced inflammatory infiltration. Both low-dose and high-dose 6-Gingerol treatment markedly improved the colonic histopathological condition of CDAD mice, particularly at a dosage of 100 mg/kg/day.

Myeloperoxidase (MPO), secreted by neutrophils, monocytes, and certain macrophages, was analyzed via immunohistochemistry to assess inflammation in colonic tissues (Figures 3B,E). The MOD group exhibited a significant increase in MPO-positive inflammatory cells compared to the CON group, indicating elevated MPO expression in CDAD mice (*P* < 0.01). 6-Gingerol treatment significantly inhibited MPO expression in CDAD mice (*P* < 0.05), comparable to the FID group.

RT-qPCR analysis was used to evaluate the expression of the pro-inflammatory cytokines TNF-α and IL-1β in colonic tissues (Figures 3C,D). Post-modeling, the MOD group showed significantly elevated expression of TNF-α and IL-1β. Treatment with 6-Gingerol significantly reduced the expression of these cytokines, with the GH group showing levels closer to the CON group and comparable to the FID group, indicating potent anti-inflammatory effects of 6-Gingerol on CDAD-induced colonic inflammation.

These findings demonstrate that high-dose 6-Gingerol (100 mg/kg/day) is more effective than low-dose (50 mg/kg/day) in alleviating CDAD symptoms, reducing *C. difficile* load (*P* < 0.001), and mitigating colonic inflammation. Therefore, subsequent studies focused on the high-dose treatment group.

### 3.4 6-Gingerol enhances intestinal barrier integrity in CDAD mice

**FIGURE 4:**
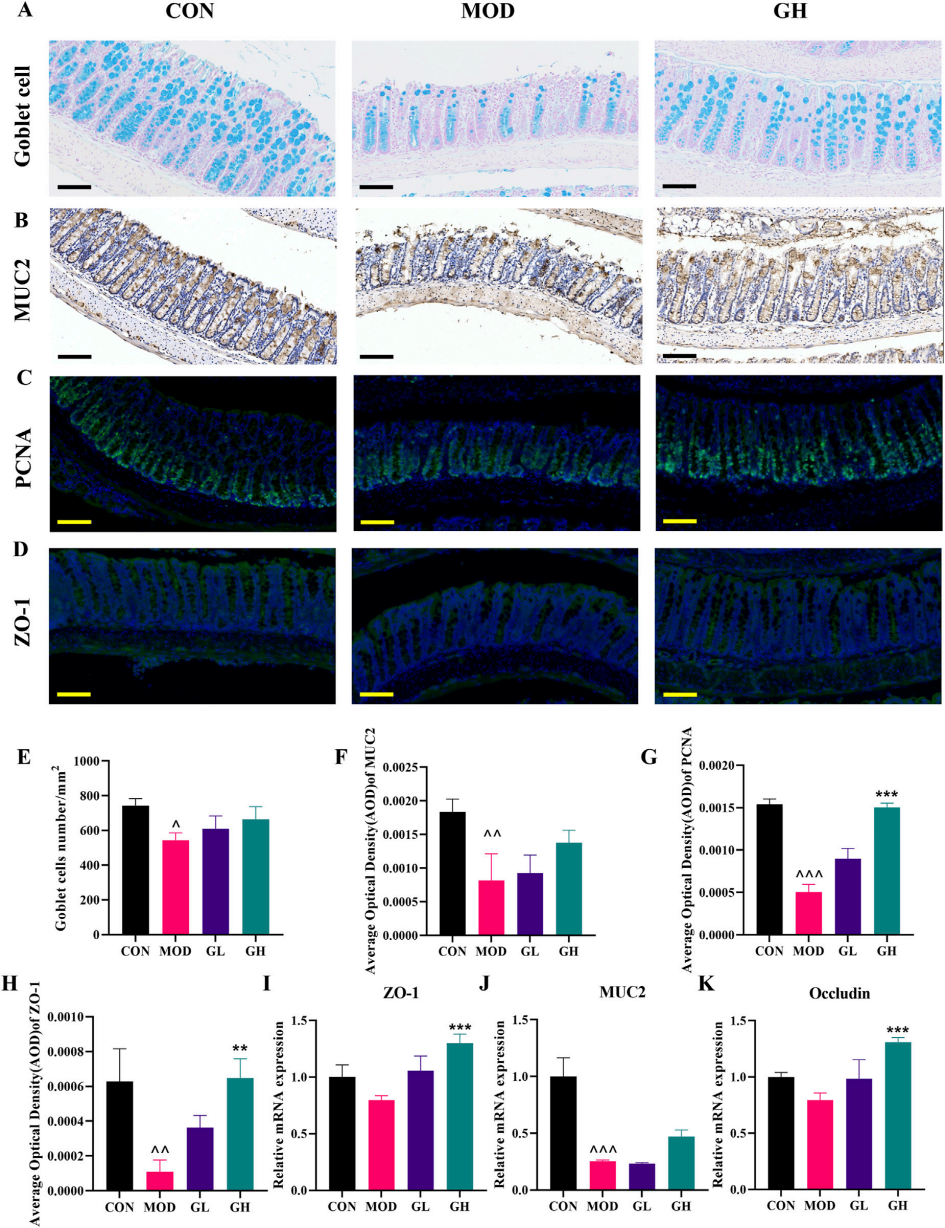
Restorative effects of 6-Gingerol on intestinal barrier integrity in CDAD mice. **(A)** Alcian blue staining for goblet cells in each group. **(B)** Immunohistochemical staining for MUC2 in colonic tissues. **(C)** Immunofluorescence staining for PCNA and **(D)** ZO-1 in colonic tissues. **(E–H)** Quantitative analysis of goblet cell numbers **(E)**, semi-quantitative analysis of MUC2 protein expression **(F)**, semi-quantitative analysis of PCNA expression **(G)**, and semi-quantitative analysis of ZO-1 expression **(H)**. **(I–K)** RT-qPCR analysis of ZO-1 **(I)**, MUC2 **(J)**, and Occludin **(K)** gene expression. Original magnification: 200x, scale bar: 100 μm. Values are expressed as mean ± standard deviation, n = 3/group. Statistical comparisons were performed using one-way ANOVA. * indicates a significant difference between the MOD and GH groups, *P < 0.05, **P < 0.01, ***P < 0.001. ^ indicates a significant difference between the MOD and CON groups, ^*P* < 0.05, ^ ^*P* < 0.01, ^ ^ ^*P* < 0.001.

To investigate changes in intestinal barrier function associated with CDAD, we assessed the number of goblet cells, the expression of proliferating cell nuclear antigen (PCNA), mucin (MUC2), tight junction protein ZO-1, and integral membrane protein Occludin in colonic tissues (Figure 4). Alcian blue staining revealed a significant decrease in goblet cell numbers in the MOD group, which was markedly restored following 6-Gingerol intervention (Figures 4A,E).

Immunofluorescence was employed to detect PCNA and ZO-1 expression, and immunohistochemistry was used for MUC2 expression. The MOD group exhibited significantly reduced PCNA levels compared to the CON group, whereas 6-Gingerol treatment significantly restored PCNA expression (Figures 4C,G). Similarly, MUC2 expression in the MOD group was significantly lower than in the CON group, but it was significantly elevated in the GH treatment group (Figures 4B,F), a finding further corroborated by RT-qPCR at the gene level (Figure 4J). ZO-1 expression followed a similar trend to MUC2, with significant reductions in the MOD group and significant increases in the GH group following 6-Gingerol treatment (Figures 4D,H). RT-qPCR results for ZO-1 were consistent with immunofluorescence findings (Figure 4I). Additionally, Occludin expression was significantly enhanced by 6-Gingerol treatment, as confirmed by RT-qPCR (Figure 4K).

These results indicate that 6-Gingerol significantly ameliorates CDAD-induced intestinal barrier dysfunction. This improvement might be achieved through restoring goblet cell numbers, promoting PCNA and Occludin expression, and enhancing the expression of MUC2 and ZO-1, thereby facilitating intestinal barrier repair and contributing to the therapeutic efficacy against CDAD.

### 3.5 6-Gingerol modulates gut microbiota in CDAD mice

**FIGURE 5:**
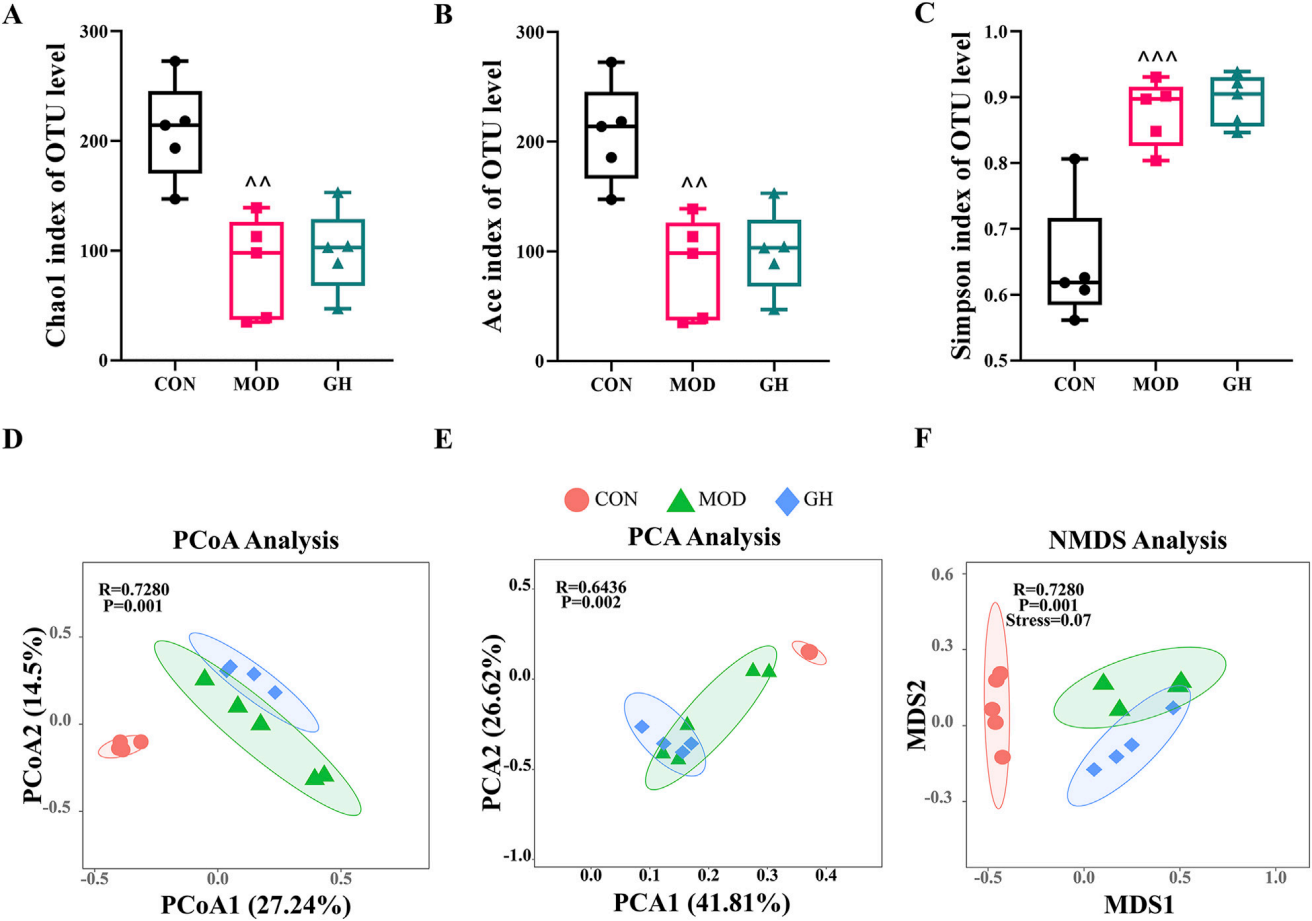
The impact of 6-Gingerol on gut microbiota diversity. **(A–C)** Alpha diversity indices: Chao1 **(A)**, ACE **(B)**, and Simpson **(C)**. **(D–F)** Beta diversity analyses: PCA **(D)**, PCoA **(E)**, and NMDS **(F)**. Values are expressed as mean ± standard deviation, n = 5/group. Alpha diversity was analyzed using one-way ANOVA, and beta diversity was evaluated by PERMANOVA based on Bray–Curtis distances. ^ indicates a significant difference between the MOD and CON groups, ^*P* < 0.05, ^ ^*P* < 0.01, ^ ^ ^*P* < 0.001.

The overuse or inappropriate use of antibiotics disrupts the gut microbiota, compromising its barrier function and facilitating *C. difficile* colonization. Based on the therapeutic effects observed in our initial results, we further investigated the impact of 6-Gingerol on the gut microbiota to explore its potential mechanisms. Firstly, we analyzed the diversity of the gut microbiota post-6-Gingerol intervention. Alpha diversity, reflecting within-group diversity, was assessed using six key indices: ACE, Chao1, Fisher, Coverage, Simpson, and Observed. ACE and Chao1 indices elucidated species richness within communities. The Fisher index combined richness and evenness for a comprehensive diversity assessment. The Observed index indicated the number of detected species, while the Simpson index measured species diversity, and the Coverage index estimated sample coverage completeness. Post-modeling, gut microbiota richness and diversity significantly declined. 6-Gingerol treatment showed trends towards restoration in the GH group, with indices approaching those of the CON group (Figures 5A,B) (Supplementary Figures S1A, B). All groups exhibited over 99% Coverage, indicating sufficient sequencing depth for accurate community structure representation (Supplementary Figure S1C).

We further evaluated microbial community composition through Beta diversity analysis, employing Jaccard index-based Principal Coordinates Analysis (PCoA), Principal Component Analysis (PCA), and Non-metric Multidimensional Scaling (NMDS). These analyses indicated inter-group similarities or differences. PCoA revealed significant compositional differences between the MOD and CON groups, indicating altered gut microbiota in the MOD group. The GH group showed compositional changes post-6-Gingerol intervention compared to the MOD group. PCA and NMDS results corroborated PCoA findings, underscoring the impact of 6-Gingerol on gut microbiota composition (Figures 5E,F).

**FIGURE 6:**
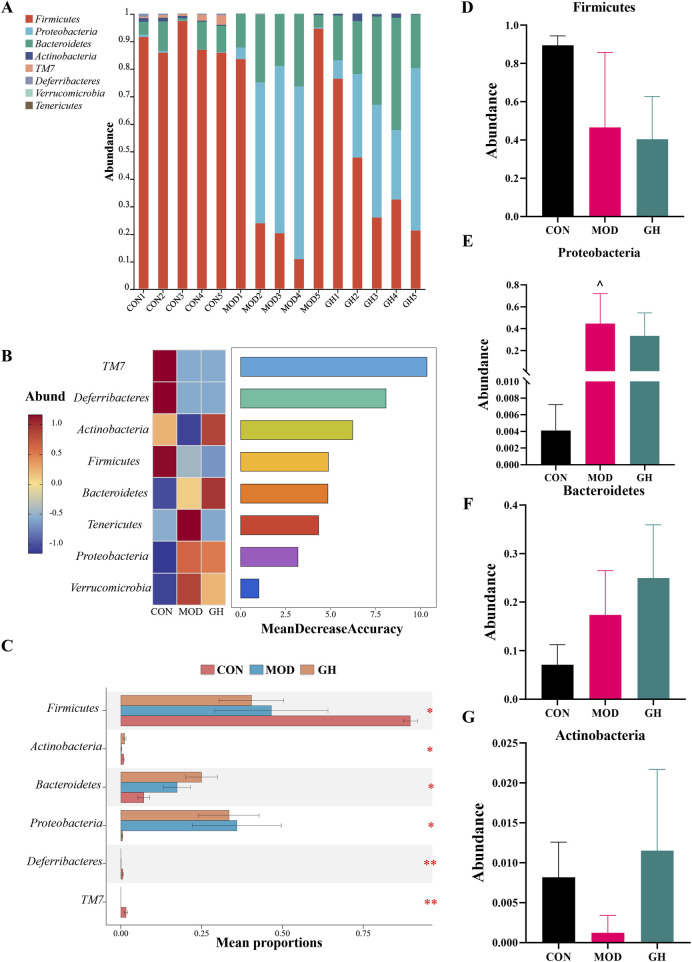
Impact of 6-Gingerol on gut microbiota at the phylum level. **(A)** Relative abundance at the phylum level. **(B)** Random forest analysis model results, ranked by Mean Decrease Accuracy for phylum-level differences. **(C)** Comparison of phylum-level microbiota composition among groups. **(D–G)** Abundance analysis: Firmicutes **(D)**, Proteobacteria **(E)**, Bacteroidetes **(F)**, Actinobacteria **(G)**. Values are expressed as mean ± standard deviation, n = 5/group. Data were analyzed using the Kruskal–Wallis test or one-way ANOVA, depending on data distribution. *P < 0.05, **P < 0.01, ***P < 0.001. ^ indicates a significant difference between the MOD and CON groups, ^*P* < 0.05, ^ ^*P* < 0.01, ^ ^ ^*P* < 0.001.

**FIGURE 7:**
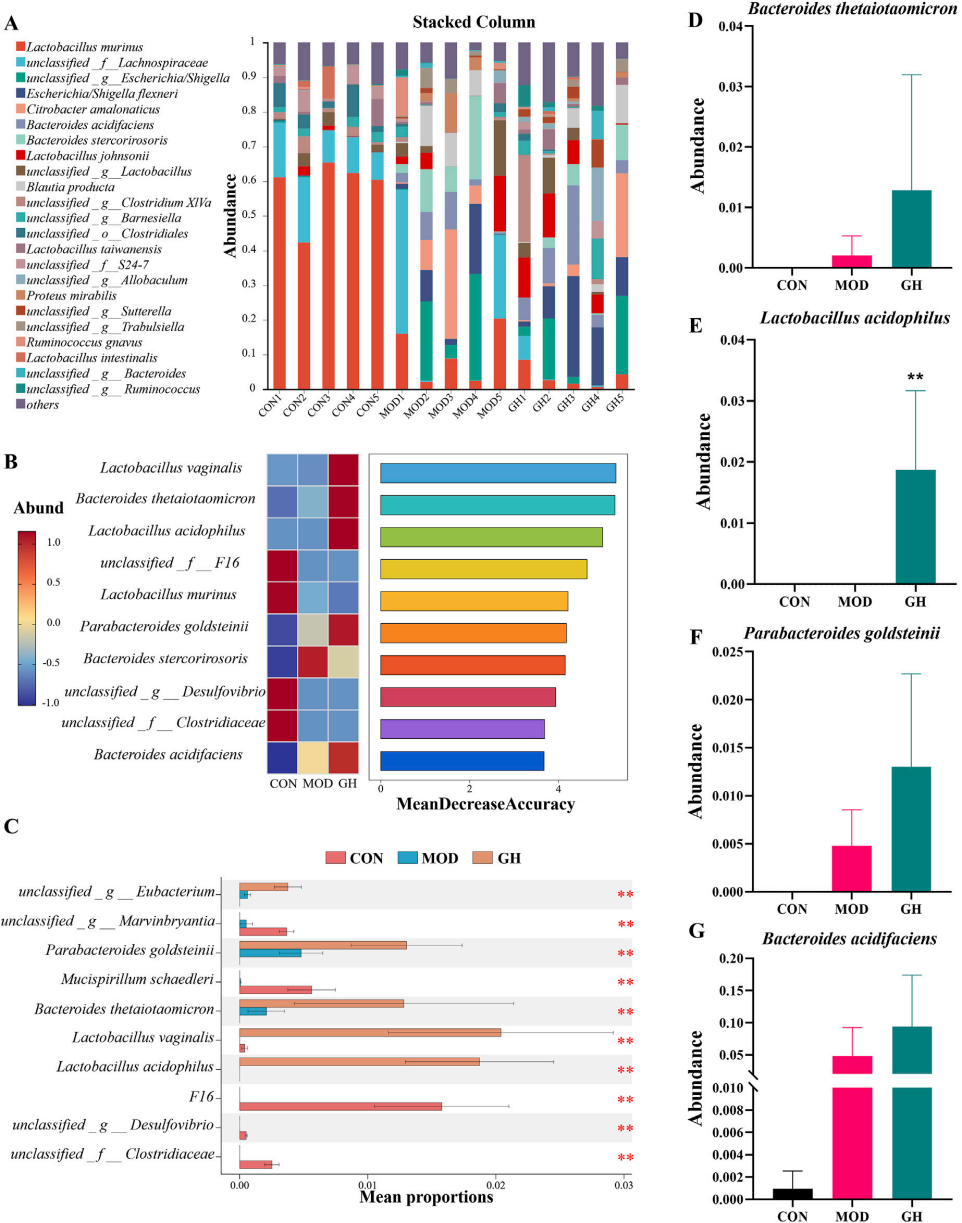
Impact of 6-Gingerol on gut microbiota at the species level. **(A)** Relative abundance at the species level. **(B)** Random forest analysis model results, ranked by Mean Decrease Accuracy for species-level differences. **(C)** Comparison of species-level microbiota composition among groups. **(D–G)** Abundance analysis: *Bacteroides thetaiotaomicron* **(D)**, *Lactobacillus acidophilus* **(E)**, *Parabacteroides goldsteinii* **(F)**, *Bacteroides acidifaciens* **(G)**. Values are expressed as mean ± standard deviation, n = 5/group. Data were analyzed using the Kruskal–Wallis test or one-way ANOVA, depending on data distribution. *P < 0.05, **P < 0.01, ***P < 0.001.

Subsequently, we analyzed the composition of gut microbiota at the phylum (Figure 6) and species (Figure 7) levels based on OTU absolute abundance and annotation information. Firmicutes, Proteobacteria, and Bacteroidetes were the dominant phyla. Post-modeling, Firmicutes decreased, while Proteobacteria and Bacteroidetes increased. 6-Gingerol intervention showed a trend towards restoring Proteobacteria proportions to CON group levels. Using random forest algorithms, we identified biomarkers, ranked by Mean Decrease Accuracy (Figure 6B). Random forest and Stamp analyses indicated increased Actinobacteria, Tenericutes, and Verrucomicrobia in the GH group, suggesting partial restoration of microbiota at the phylum level post-6-Gingerol treatment.

At the species level, the most abundant taxa were *Lactobacillus murinus*, *unclassified_f__Lachnospiraceae*, and *unclassified_g__Escherichia/Shigella* (Figure 7A). Modeling significantly reduced *L. murinus* abundance, with no substantial change in the GH group compared to the MOD group. *unclassified_f__Lachnospiraceae* abundance remained unchanged in the MOD group but decreased in the GH group. *Unclassified_g__Escherichia/Shigella* significantly increased in the MOD group, with the GH group showing trends towards CON group levels. Using random forest algorithms, we identified potential biomarker taxa, with top 10 ranked by importance (Figure 7B). Stamp analysis identified differences among groups (Figure 7C). Notably, *Lactobacillus acidophilus*, *Bacteroides thetaiotaomicron*, *Parabacteroides goldsteinii*, and *Bacteroides acidifaciens* were elevated in the GH group (Figures 7D–G). These taxa are closely associated with short-chain fatty acids (SCFAs) production (Modasia et al., 2020; Takei et al., 2020; Vemuri et al., 2019; Wang et al., 2021). To determine whether changes in gut microbiota influence SCFAs production, we conducted targeted SCFAs analysis.

### 3.6 6-Gingerol enhances short-chain fatty acids production in CDAD mice

Short-chain fatty acids (SCFAs) have a direct inhibitory effect on the growth of *C*. *difficile* and alleviate CDAD symptoms by reducing intestinal inflammation, increasing tight junction protein expression, and modulating the host immune response (Ouyang et al., 2022). Using ultra-performance liquid chromatography-mass spectrometry (UPLC-MS), we successfully quantified SCFAs in fecal samples from different groups of mice. The SCFAs analyzed, along with their chemical formulas, are presented in Supplementary Figure S4. Total ion chromatograms (TIC) for standards and fecal samples are shown in Supplementary Figures S6A–E, respectively. Standard curves for each SCFAs are depicted in Supplementary Figure S5.

**FIGURE 8:**
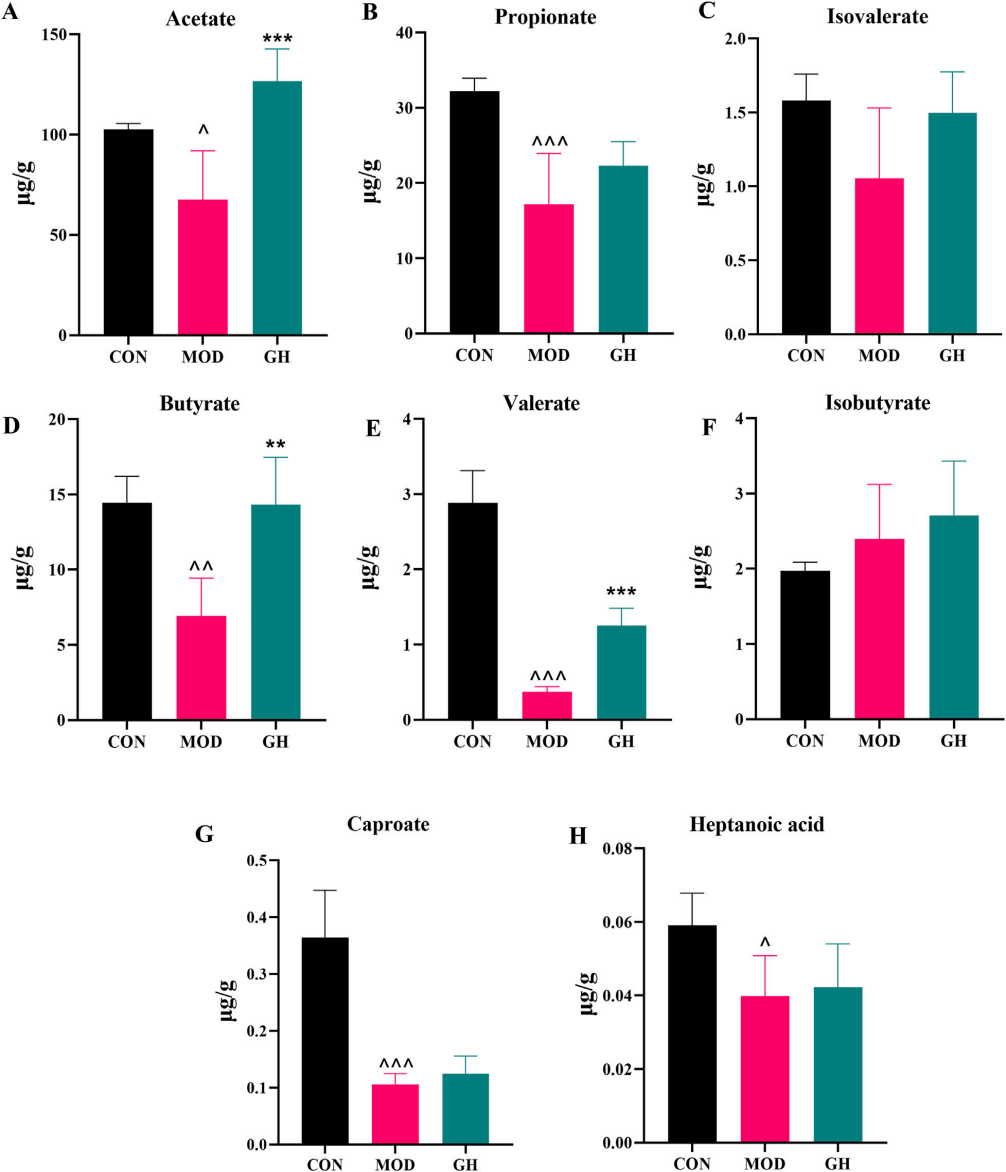
The restorative effects of 6-Gingerol on SCFAs levels in mice. **(A–H)** Analysis of SCFAs content in fecal samples from three groups: acetate **(A)**, propionate **(B)**, isovalerate **(C)**, butyrate **(D)**, valerate **(E)**, isobutyrate **(F)**, caproate **(G)**, and heptanoic acid **(H)**. Values are expressed as mean ± standard deviation, n = 5/group. Statistical comparisons were performed using one-way ANOVA. * indicates a significant difference between the MOD and GH groups, *P < 0.05, **P < 0.01, ***P < 0.001. ^ indicates a significant difference between the MOD and CON groups, ^*P* < 0.05, ^ ^*P* < 0.01, ^ ^ ^*P* < 0.001.

The results (Figure 8) indicated that, except for isovalerate, the levels of all detected SCFAs were significantly reduced in the MOD group compared to the CON group. Following 6-Gingerol treatment, there was a significant increase in acetate (*P* < 0.001) (Figure 8A), butyrate (*P* < 0.01) (Figure 8D), and valerate (*P* < 0.001) (Figure 8E) levels compared to the MOD group. Other SCFAs also showed notable increases. Notably, acetate, butyrate, and valerate, which exhibited significant differences from the MOD group, have been directly linked to CDAD recovery. Thus, we propose that the restorative effects of 6-Gingerol on CDAD are closely associated with its ability to elevate SCFAs levels.

## 4 Discussion

The increasing incidence of *C. difficile*-associated diarrhea (CDAD) worldwide is primarily driven by the widespread use of broad-spectrum antibiotics. Due to its high recurrence rate, strong transmissibility, and treatment resistance, CDAD has become a serious public health challenge (Martin et al., 2016). Therefore, a deeper understanding of CDAD pathogenesis and potential therapeutic strategies has become a research priority. Following *C. difficile* infection, the composition of the normal gut microbiota is disrupted, intestinal metabolites are altered, and *C. difficile* in the colon produces toxins TcdA and TcdB, leading to severe immune responses, intestinal inflammation, and disruption of the intestinal barrier (Leffler and Lamont, 2015).

This study consists of two parts. The first part involves an *in vitro* antibacterial assay aimed at verifying whether 6-Gingerol has a direct inhibitory effect on *C. difficile*. The second part comprises an *in vivo* animal experiment, which investigates the effects of 6-Gingerol on intestinal inflammation, barrier function, microbiota composition, and short-chain fatty acids (SCFAs) levels from a pharmacodynamic perspective.

The *in vitro* antibacterial assay results showed that 6-Gingerol exhibited inhibitory effects on the growth of *C. difficile*. When the concentration of 6-Gingerol reached 61.99 μM, the growth inhibition rate of *C. difficile* reached 50% (IC50), while complete inhibition was achieved at 173.3 μM (MIC) (Figure 1). These results suggest that 6-Gingerol exerts strong direct antibacterial activity *in vitro*. However, in the *in vivo* experiments, the protective effects of 6-Gingerol against CDAD may not rely solely on its direct antibacterial activity but rather involve multiple regulatory mechanisms.

The pharmacodynamic evaluation of the *in vivo* animal experiments demonstrated that high-dose 6-Gingerol significantly reduced the *C. difficile* load in the colon and alleviated CDAD symptoms in mice, consistent with the *in vitro* results. Further analysis revealed that 6-Gingerol effectively alleviated colonic mucosal infiltration of inflammatory cells, epithelial cell shedding, and goblet cell loss while significantly restoring the expression of intestinal barrier-related proteins such as MUC2 and ZO-1. Overall, 6-Gingerol exhibited significant efficacy in mitigating *C. difficile*-induced intestinal damage and inflammation, aligning with previous studies reporting its strong antibacterial and anti-inflammatory properties (Ouyang et al., 2022).

Previous studies have emphasized that maintaining gut microbiota homeostasis plays a crucial role in resisting pathogenic infections, suggesting that modulating microbiota composition could be a potential therapeutic strategy for CDAD (Buckley et al., 2022; Herrera et al., 2021; Smits et al., 2016). However, antibiotic treatment for CDAD may further disrupt microbiota balance and exacerbate antibiotic resistance issues. Fecal microbiota transplantation (FMT), which involves transferring gut microbiota from healthy donors to patients, has gained increasing attention as an effective treatment for recurrent CDAD (Hvas et al., 2019).

To further explore the therapeutic mechanisms of 6-Gingerol, we analyzed its impact on gut microbial composition. 16S rRNA gene sequencing revealed that 6-Gingerol partially restored the dysbiotic microbiota structure induced by CDAD (Figures 5–7; Supplementary Figures S2, S3). At the species level, the GH group exhibited elevated levels of *L. acidophilus*, *Bacteroides thetaiotaomicron*, *P. goldsteinii*, and *Bacteroides acidifaciens*, all of which are closely associated with SCFA production (Figures 7D–G) (Modasia et al., 2020; Takei et al., 2020; Vemuri et al., 2019; Wang et al., 2021).

Notably, *L. murinus* did not show a marked recovery following 6-Gingerol treatment. As illustrated in Figure 7A, its abundance significantly declined in the model group and remained at a low level even after high-dose intervention. Although this finding is often overlooked, it may have biological significance. The results suggest that the therapeutic effects of 6-Gingerol on CDAD are not dependent on the restoration of *L. murinus*, but rather on the enrichment of other beneficial SCFAs-producing bacteria, such as *L. acidophilus*, *Bacteroides thetaiotaomicron*, *P. goldsteinii*, and *Bacteroides acidifaciens* (Figures 7D–G). These taxa are closely associated with SCFAs biosynthesis and the maintenance of gut homeostasis. The elevated levels of acetate, butyrate, and valerate observed in Figure 8 further support the hypothesis that gut function may be restored through compensatory mechanisms involving these microbial taxa, highlighting the functional redundancy within the gut microbiota.

In addition, the decreased abundance of *unclassified_f_Lachnospiraceae* in the GH group warrants further attention. Although its levels remained relatively unchanged in the model group, a reduction was observed following 6-Gingerol treatment, which may be attributed to microbial competition or shifts in substrate availability. Since 6-Gingerol promoted the enrichment of other SCFAs-producing bacteria, *unclassified_f_Lachnospiraceae* may have been competitively excluded. Nonetheless, considering that SCFAs levels remained stable or even increased after treatment, it is plausible that taxa such as *L. acidophilus* functionally compensated for this reduction, thereby maintaining SCFAs production and contributing to the restoration of gut function.

SCFAs are important metabolites produced by gut microbiota, with certain bacterial taxa capable of converting dietary components into SCFAs, which have been shown to play crucial roles in gut health regulation, inflammation reduction, and barrier function enhancement (Ouyang et al., 2022). UPLC-MS-based quantitative analysis of fecal SCFAs levels demonstrated that 6-Gingerol treatment significantly increased the levels of acetate, butyrate, and valerate. Previous studies have shown that *L. acidophilus* enhances butyrate, propionate, and acetate levels in mouse intestines; (Vemuri et al., 2019); *Bacteroides thetaiotaomicron* promotes acetate and propionate production; (Modasia et al., 2020) *P. goldsteinii* is positively correlated with cecal SCFA content; (Wang et al., 2021) and *Bacteroides acidifaciens* participates in fermentation, producing various beneficial SCFAs (Takei et al., 2020). Our study found that 6-Gingerol enhances the proliferation of these key bacterial taxa (Figure 7), leading to an increase in SCFA levels (Figure 8), which may be one of the key mechanisms underlying its protective effects against CDAD.

To further elucidate the biological significance of SCFAs elevation following 6-Gingerol treatment, we performed a species-level correlation heatmap analysis focusing on bacterial taxa closely associated with SCFAs production and key pharmacodynamic indicators. As shown in Supplementary Figure S7B, the abundance of known SCFAs-producing species such as *L. acidophilus* and *L. murinus* was positively correlated with fecal SCFAs levels, suggesting that 6-Gingerol may improve the intestinal metabolic environment and suppress pathogenic colonization by promoting the growth of beneficial acid-producing bacteria. Additionally, Supplementary Figure S7A demonstrated that SCFAs levels were negatively correlated with pro-inflammatory cytokines (e.g., TNF-α and IL-1β) and positively correlated with the expression of tight junction proteins (ZO-1 and Occludin), indicating a potential role of SCFAs in alleviating inflammation and enhancing intestinal barrier integrity. It is important to note, however, that these findings reflect statistical associations rather than direct causality. Further mechanistic studies involving SCFAs supplementation or inhibition are warranted to validate their functional roles. Nevertheless, these results provide clearer mechanistic insight into how 6-Gingerol may exert therapeutic effects in CDAD by modulating key metabolic bacteria and promoting SCFAs biosynthesis.

6-Gingerol, a bioactive compound derived from the medicinal plant ginger, exhibits low toxicity and holds promise as an alternative therapeutic option for CDAD (de Lima et al., 2018; Oyagbemi et al., 2010). Our findings suggest that 6-Gingerol significantly improves CDAD through multiple synergistic mechanisms. While the direct antibacterial activity of 6-Gingerol against *C. difficile* is relatively limited, its *in vivo* efficacy is likely mediated by comprehensive mechanisms, primarily involving gut microbiota modulation and host immune enhancement. Specifically, 6-Gingerol promotes the proliferation of *L. acidophilus, Bacteroides thetaiotaomicron*, and other related bacteria, competitively inhibiting *C. difficile* colonization while increasing SCFA levels (e.g., acetate, butyrate, and valerate), thereby suppressing *C. difficile* load. Additionally, 6-Gingerol enhances the expression of tight junction proteins such as ZO-1 and Occludin, improving intestinal barrier function, reducing bacterial translocation and toxin infiltration, and mitigating CDAD-associated inflammation, further weakening the survival advantage of *C. difficile*.

**FIGURE 9:**
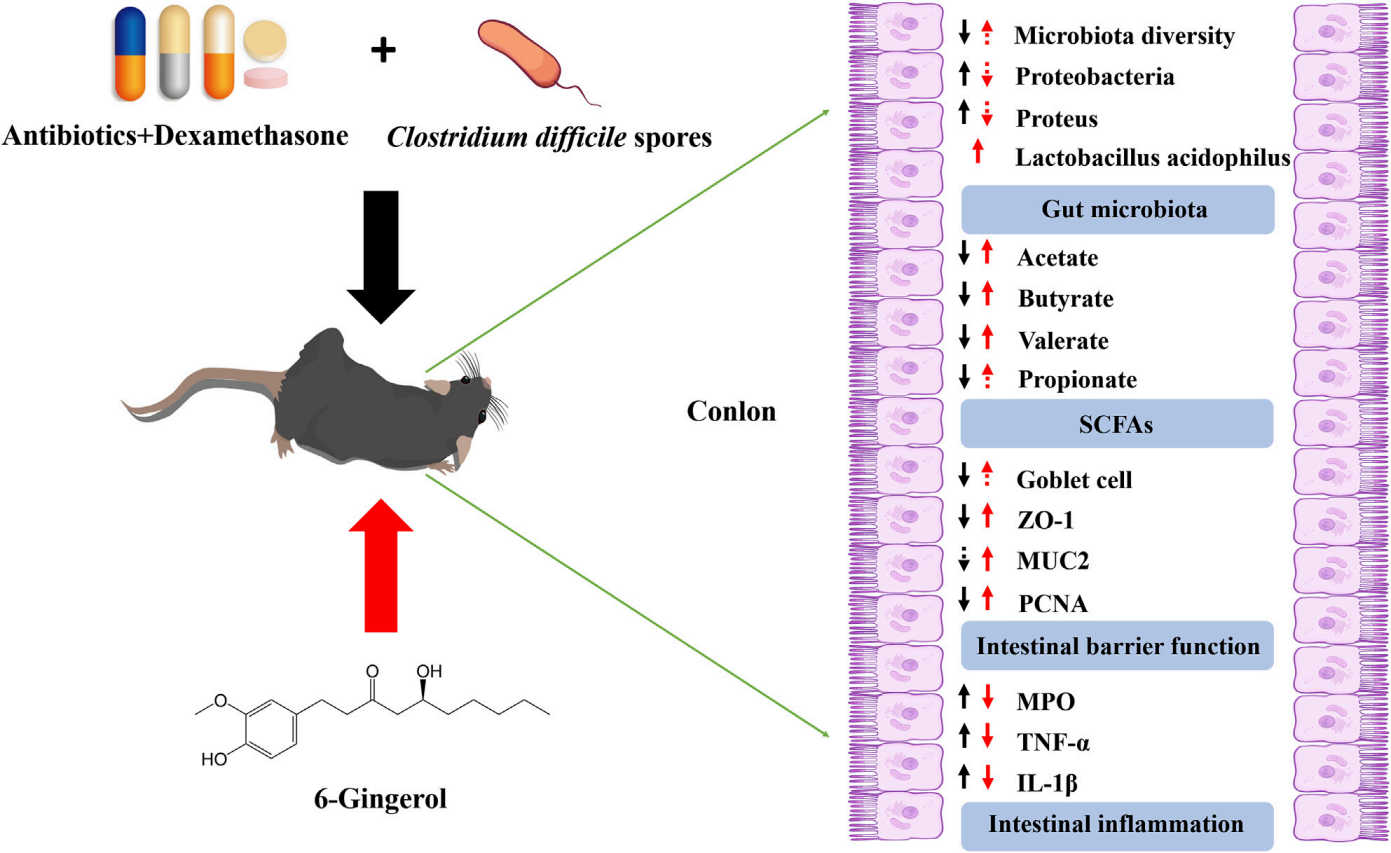
The overview effects of 6-gingerol on intestinal microbiota, short-chain fatty acids, intestinal barrier and intestinal inflammation.

In summary, the potential therapeutic value of 6-Gingerol in CDAD management stems from its direct and indirect antibacterial effects, modulation of gut microbiota, restoration of SCFA levels, enhancement of intestinal barrier function, and anti-inflammatory properties (Figure 9). These multifaceted mechanisms provide a strong foundation for further clinical investigations. However, this study has certain limitations. The findings from animal models, particularly those from gut microbiota sequencing and SCFA quantification, may not be directly applicable to humans due to inherent physiological differences. Therefore, while this study provides critical insights into the pharmacological effects and potential mechanisms of 6-Gingerol, further clinical trials are warranted before its clinical application as a CDAD treatment.

## Data Availability

The data generated in this study have been deposited in the NCBI Sequence Read Archive (SRA) under BioProject accession number PRJNA1305174 and are publicly available at https://www.ncbi.nlm.nih.gov/bioproject/PRJNA1305174.
