# Correction: Preclinical evaluation of 6-Gingerol in modulating gut microbiota and SCFAs to mitigate *Clostridium difficile*-associated diarrhea in mice

**DOI:** 10.3389/fchem.2026.1785599

**Published:** 2026-01-23

**Authors:** Fu-Zhi Ma, Lin Zhu, Meng Li, Ze-Wei Tang, Xiao-Hong Yu, Cong-En Zhang, Zhi-Jie Ma

**Affiliations:** 1 Department of Pharmacy, Beijing Ditan Hospital, Capital Medical University, Beijing, China; 2 Department of Pharmacy, Beijing Friendship Hospital, Capital Medical University, Beijing, China; 3 College of Traditional Chinese Medicine, Yunnan University of Chinese Medicine, Kunming, Yunnan, China

**Keywords:** 6-Gingerol, *Clostridium difficile*-associated diarrhea, pharmacodynamic evaluation, gut microbiota, short-chain fatty acids (SCFAs)

The funder National Natural Science Foundation of China, grant number 82474163 to Zhijie Ma was erroneously omitted. The correct **Funding** statement reads:

The author(s) declared that financial support was received for this work and/or its publication. This work was supported by the National Key R&D Program of China (2023YFC2308200), the National Natural Science Foundation of China (No. 82474163), the National Natural Science Foundation of China (No. 82374053), and the Beijing Hospitals Authority High-Level Public Health Technical Talent Development Project (Code: Academic Leader-02-28). This support significantly contributed to the success of our research.

The original article has been updated.

