# Supplementary material for: Preclinical evaluation of 6-Gingerol in modulating gut microbiota and SCFAs to mitigate Clostridium difficile-associated diarrhea in mice
Source: Front Chem. 2025 Aug 28;13:1635781. doi: 10.3389/fchem.2025.1635781 (PMC12423060; doi:10.3389/fchem.2025.1635781)
Supplement: Supplementary file 1 [file DataSheet1.docx]

Supplementary Material

**Preclinical evaluation of 6-Gingerol in modulating gut microbiota and SCFAs to mitigate *Clostridium difficile*-associated diarrhea in mice Fu-zhi Ma ^1^, Lin Zhu ^1^, Meng Li ^3^, Ze-wei Tang ^2^, Xiao-hong Yu^3^, Cong-en Zhang ^3*^, Zhi-jie Ma ^1*^**

^1^ [Department of Pharmacy](https://xueshu.baidu.com/s?wd=author:(Department of Pharmacy) &tn=SE_baiduxueshu_c1gjeupa&ie=utf-8&sc_f_para=sc_hilight=person" \t "D:/C盘文件/Desktop/Antimicrobial%20agents%20and%20chemotherapy%20投稿/新提交Antimicrobial%20agents%20and%20chemotherapy/_blank), [Beijing Ditan Hospital](https://xueshu.baidu.com/s?wd=author:(Beijing%20Ditan%20Hospital)%20&tn=SE_baiduxueshu_c1gjeupa&ie=utf-8&sc_f_para=sc_hilight=person), [Capital Medical University](https://xueshu.baidu.com/s?wd=author:(Capital%20Medical%20University)%20&tn=SE_baiduxueshu_c1gjeupa&ie=utf-8&sc_f_para=sc_hilight=person), 100015, Beijing, China.

^2^ College of Traditional Chinese Medicine, Yunnan University of Chinese Medicine, Kunming, 650500, Yunnan, China.

^3^ Department of Pharmacy, Beijing Friendship Hospital, Capital Medical University, 100050, Beijing, China.

***Correspondence:**

Zhi-jie Ma, Beijing Ditan Hospital, Capital Medical University, No. 8, Jingshun East Street, 100015, Beijing, China. E-mail: mazj2021@163.com.

Cong-en Zhang, Beijing Friendship Hospital, Capital Medical University, No. 95, Yongan Road, 100050, Beijing, China. E-mail: zce820@163.com.

**1. Regulation of gut microbiota in CDAD mice by 6-Gingerol treatment**


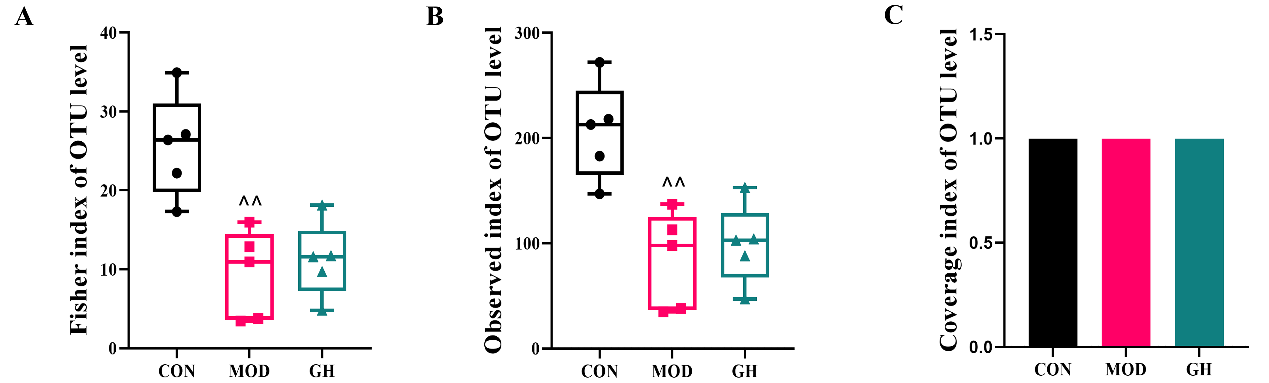


**Supplementary Figure 1. 6-Gingerol treatment alters gut microbiota diversity in CDAD mice.** Alpha-diversity analysis of different microbial abundances is based on the Fisher index at the OTU level (A), the Observed index (B), and the Coverage index (C). Values are expressed as mean ± standard deviation, n=5/group. Statistical comparisons were performed using the one-way ANOVA. ^ indicates a significant difference between the MOD and CON groups, ^^*P* < 0.01.


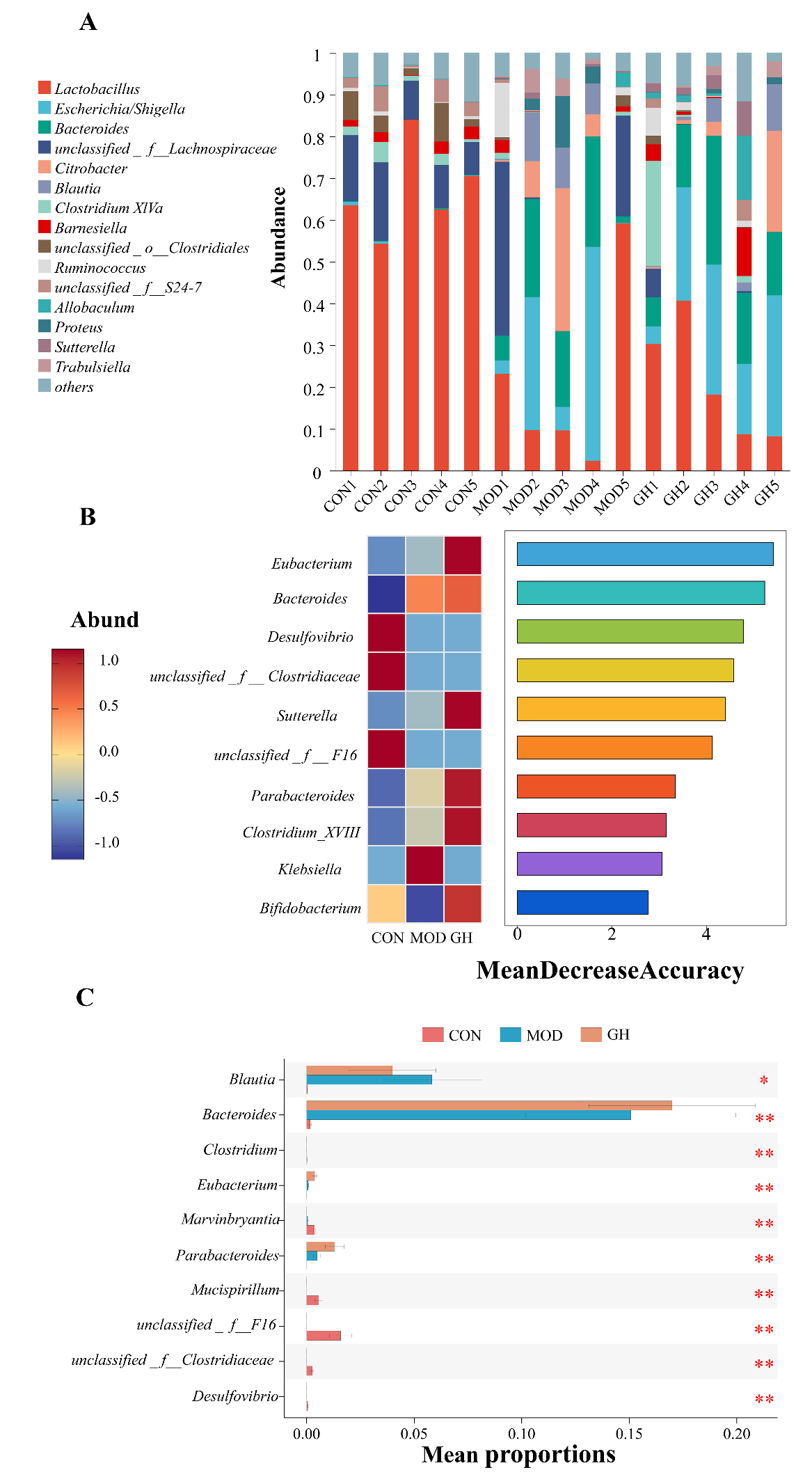


**Supplementary Figure 2. Impact of 6-Gingerol on the composition of gut microbiota at the genus level in mice.** (A) Relative abundance of gut microbiota at the genus level. (B) Model prediction results using random forest algorithm for genus-level gut microbiota, ranked by average decrease in accuracy (x-axis) to identify group differences. (C) Comparison of genus-level microbiota composition among the three groups. Values are expressed as mean ± standard deviation, n=5/group. Statistical comparisons were performed using the Kruskal–Wallis test. *P* < 0.05 *, *P* < 0.01 **.


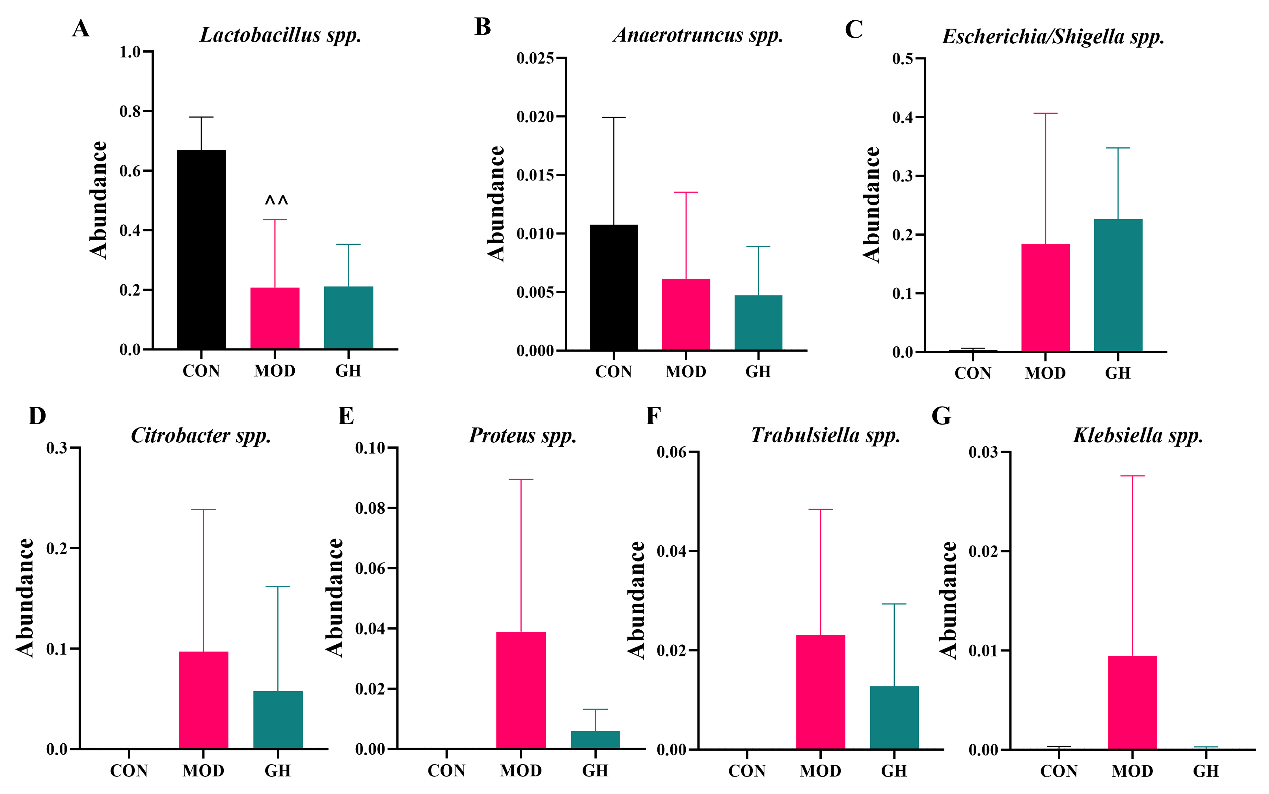


**Supplementary Figure 3. Effect of 6-Gingerol on specific genera of the gut microbiota in mice.** (A-G) Abundance analysis of *Lactobacillus spp.* (A), *Anaerotruncus spp.* (B), *Escherichia/Shigella spp.* (C), *Citrobacter spp.* (D), *Proteus spp.* (E), *Trabulsiella spp.* (F), *Klebsiella spp.* (G). Values are expressed as mean ± standard deviation, n=5/group. Statistical comparisons were performed using the one-way ANOVA. ^ indicates a significant difference between the MOD and CON groups, ^^*P* < 0.01.

**2. Targeted quantification of short-chain fatty acids (SCFAs)**

**2.1 Sample solution preparation**

Fecal samples were thawed at 4°C, and approximately 50 mg was weighed and mixed with 0.5 mL of water. After sonication for 10 minutes and vortex extraction for 30 minutes, 0.5 mL of methanol was added for an additional 30 minutes of vortex extraction. The mixture was centrifuged at 12,000 rpm and 4°C for 5 minutes. A 50 µL aliquot of the supernatant was mixed with 50 µL of isotope internal standard (5 µg/mL), 50 µL of 3-nitrophenylhydrazine (3-NPH, 250 mM in 50% methanol/water), and 50 µL of EDC (150 mM in 75% methanol/water with 7.5% pyridine, methanol:water:pyridine = 69.375:23.125:7.5). The mixture was incubated at 30°C for 30 minutes for derivatization. Subsequently, 50 µL of 2,6-di-tert-butyl-p-cresol (BHT) methanol solution (2 mg/mL) and 250 µL of 75% methanol/water were added. The mixture was centrifuged again at 12,000 rpm and 4°C for 5 minutes. The supernatant (100 µL) was transferred to a sample vial for mass spectrometry analysis.

**2.2 Chromatographic conditions**

Chromatographic separation was performed using a Waters ACQUITY UPLC I-CLASS system with a BEH C8 column (2.1 mm internal diameter × 100 mm length, 1.7 µm particle size). The mobile phases were A (water with 0.01% formic acid) and B (methanol:isopropanol = 8:2). The elution gradient is detailed in Table S1, with a flow rate of 0.3 mL/min, injection volume of 5.0 µL, and column temperature of 45°C.

**Supplementary Table 1. HPLC Gradient Elution Conditions**

| Time/min | Mobile phase | |
| --- | --- | --- |
|  | A（v%） | B（v%） |
| 0 | 95 | 5 |
| 2 | 85 | 15 |
| 9 | 45 | 55 |
| 10 | 0 | 100 |
| 11 | 0 | 100 |
| 11.1  13 | 95  95 | 5  5 |

**2.3 Mass spectrometry conditions**

Mass spectrometry analysis was performed using a Waters XEVO TQ-S Micro triple quadrupole mass spectrometer. The ion source voltage was set at 3.0 kV with a source temperature of 150°C, desolvation temperature of 450°C, desolvation gas flow rate of 1000 L/h, and cone gas flow rate of 10 L/h.

**2.4 Targeted data processing**

Targeted data peak areas were calculated using TargetLynx quantification software, with a retention time tolerance of 15 seconds. Quantitative results were obtained using standard curve methods.


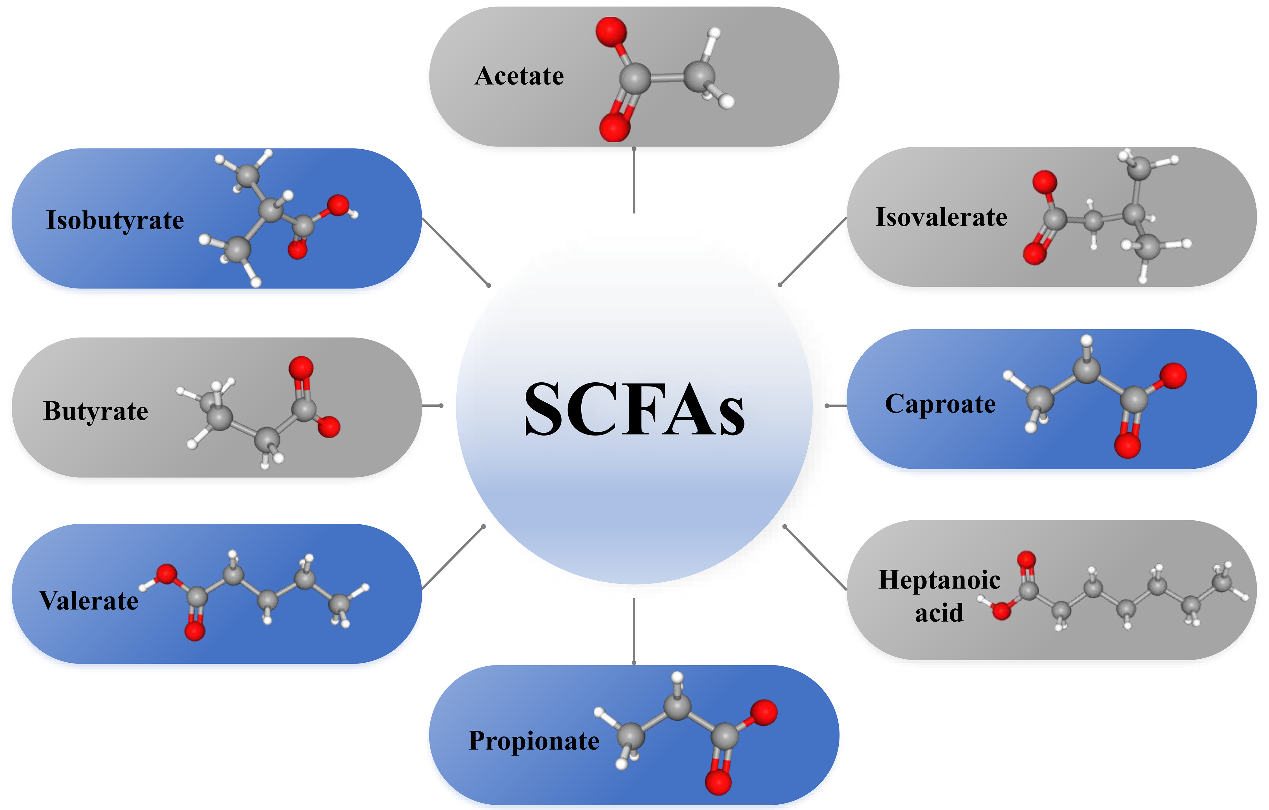


**Supplementary Figure 4. Chemical structures of targeted short-chain fatty acids.**


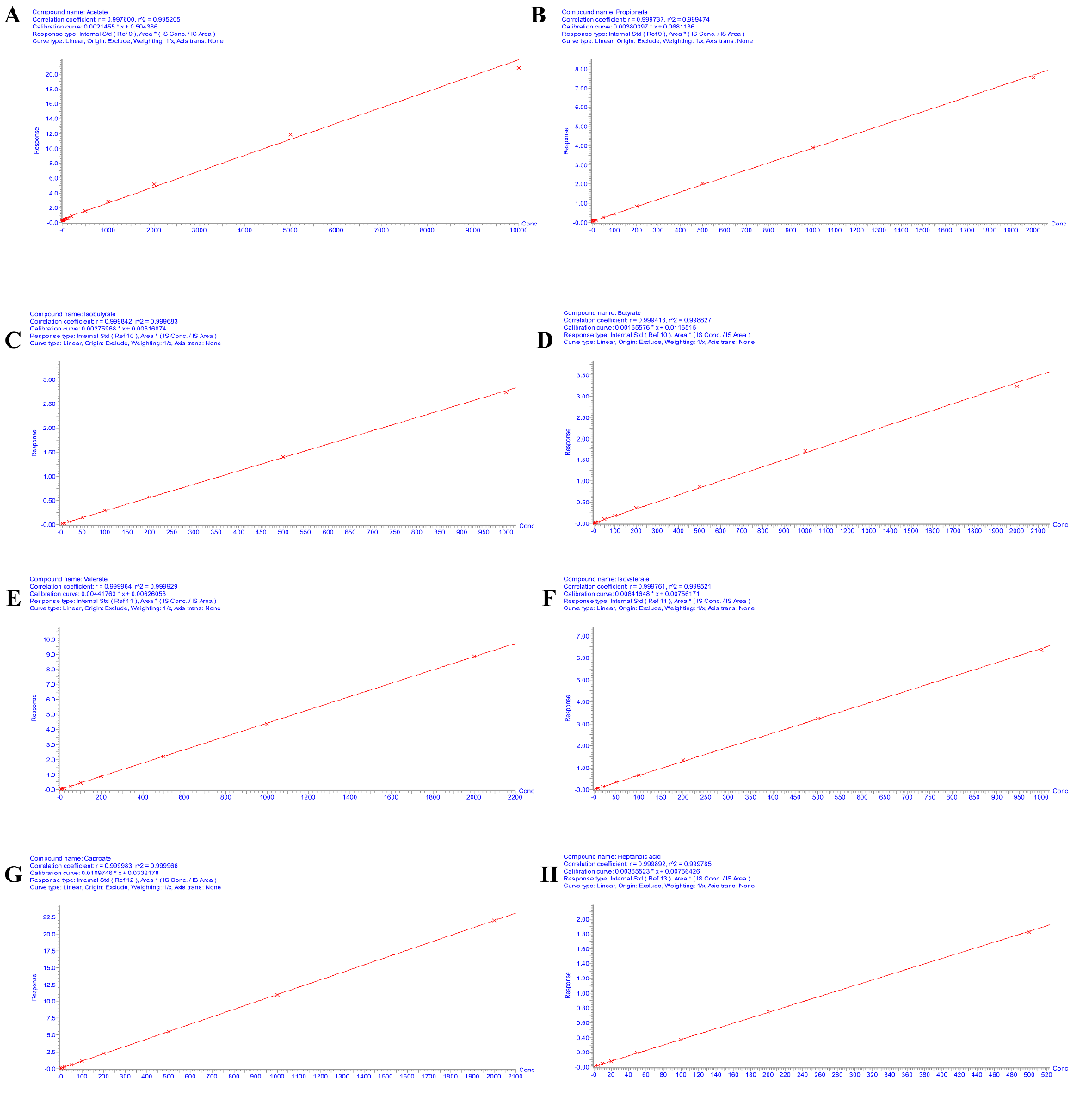


**Supplementary Figure 5. Standard curves of the detected SCFAs:** Acetate (A), Propionate (B), Isobutyrate (C), Butyrate (D), Valerate (E), Isovalerate (F), Caproate (G), Heptanoic acid (H).


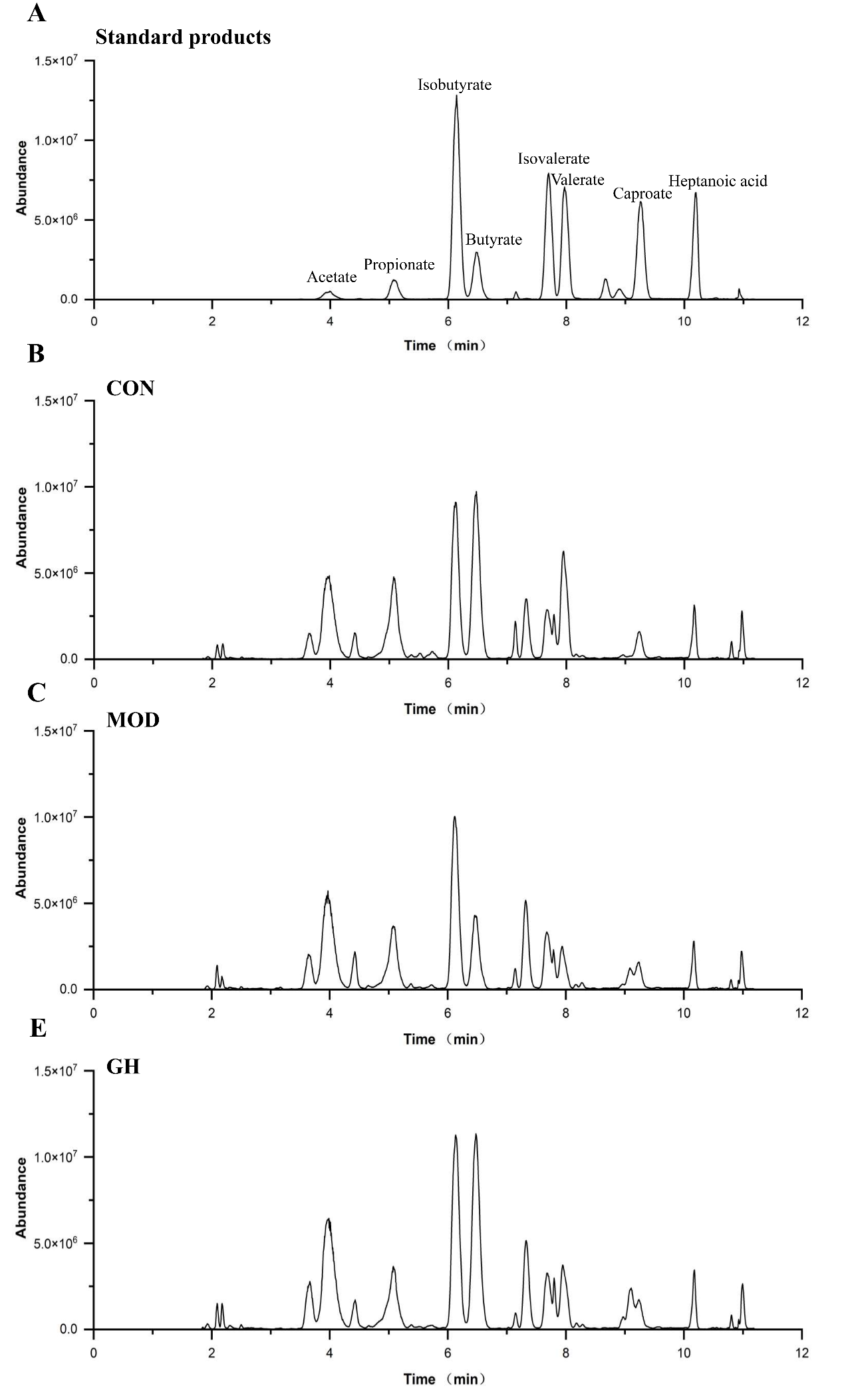


**Supplementary Figure 6. Total ion chromatograms (TIC) of standards and samples from each group:** Standards (A), CON group (B), MOD group (C), GH group (D).


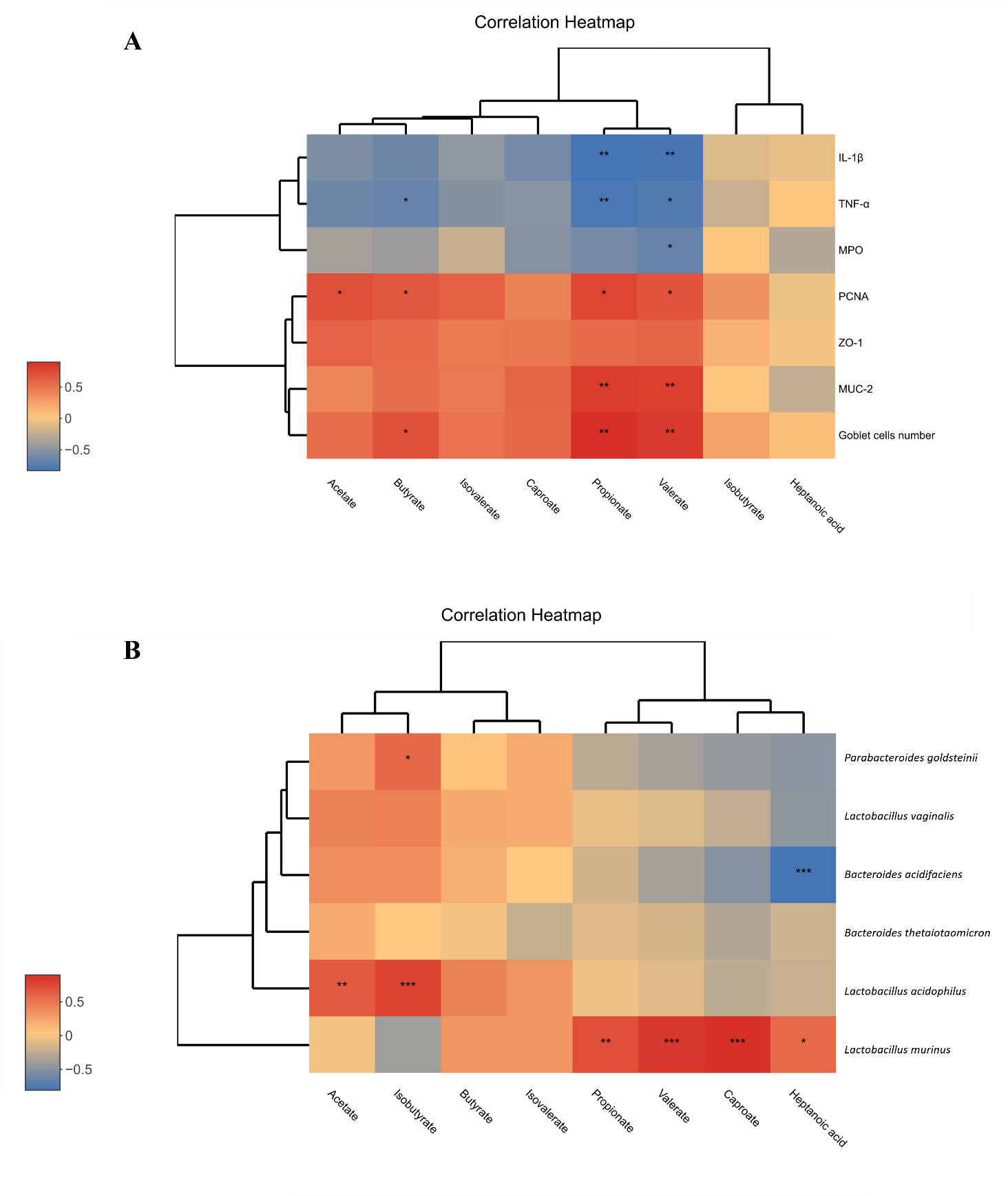


**Supplementary Figure 7. Correlation heatmaps showing associations among microbial species, SCFAs levels, and pharmacodynamic parameters:** SCFAs levels vs. inflammatory and barrier markers (A), SCFAs-producing species vs. SCFAs concentrations (B).
